# HIV-1 and Amyloid Beta Remodel Proteome of Brain Endothelial Extracellular Vesicles

**DOI:** 10.3390/ijms21082741

**Published:** 2020-04-15

**Authors:** Ibolya E. András, Brice B. Sewell, Michal Toborek

**Affiliations:** Department of Biochemistry and Molecular Biology, University of Miami School of Medicine, Miami, FL 33136-1019, USA; IAndras@med.miami.edu (I.E.A.);

**Keywords:** HIV-1, amyloid beta, extracellular vesicles, blood–brain barrier

## Abstract

Amyloid beta (Aβ) depositions are more abundant in HIV-infected brains. The blood–brain barrier, with its backbone created by endothelial cells, is assumed to be a core player in Aβ homeostasis and may contribute to Aβ accumulation in the brain. Exposure to HIV increases shedding of extracellular vesicles (EVs) from human brain endothelial cells and alters EV-Aβ levels. EVs carrying various cargo molecules, including a complex set of proteins, can profoundly affect the biology of surrounding neurovascular unit cells. In the current study, we sought to examine how exposure to HIV, alone or together with Aβ, affects the surface and total proteomic landscape of brain endothelial EVs. By using this unbiased approach, we gained an unprecedented, high-resolution insight into these changes. Our data suggest that HIV and Aβ profoundly remodel the proteome of brain endothelial EVs, altering the pathway networks and functional interactions among proteins. These events may contribute to the EV-mediated amyloid pathology in the HIV-infected brain and may be relevant to HIV-1-associated neurocognitive disorders.

## 1. Introduction

HIV-infected brains tend to have enhanced amyloid beta (Aβ) deposition [1,2,3,4,5,6], mostly in the perivascular space [3,7,8,9]. Indeed, the blood–brain barrier (BBB) is thought to be a key player in the brain’s Aβ homeostasis [10]. It is now widely accepted that extracellular vesicles (EVs) may also be important in Aβ pathology [11,12,13,14,15,16,17]. Our earlier work has shown that HIV can increase the release of brain endothelial EVs and alter EV-Aβ levels. Moreover, brain endothelial cell-derived EVs can transfer Aβ to other cells of the neurovascular unit [18]. EVs carry specific cargo molecules, including a complex set of proteins, which can be transferred to the neighboring cells and affect their biology. Some of these proteins are on the EV surface. The surface proteins may allow for selective EV uptake by the recipient cells, like in the case of receptor-mediated endocytosis. Total proteomics can give detailed information on the EV protein cargo overall. Surface proteomics could indicate the “address” of a targeted delivery, while total proteomics would represent the delivered “package.”

In this work, we investigated how exposure to HIV, alone and together with Aβ, impacts the surface and total proteomic landscape of EVs from human brain microvascular endothelial cells (HBMEC-EVs). By using this unbiased strategy, we obtained a complex, high-resolution insight into these changes. 

## 2. Results

### 2.1. Extracellular Vesicles from Human Brain Microvascular Endothelial Cells (HBMEC-EVs) Are Enriched with the Major EV Markers

**Figure 1:**
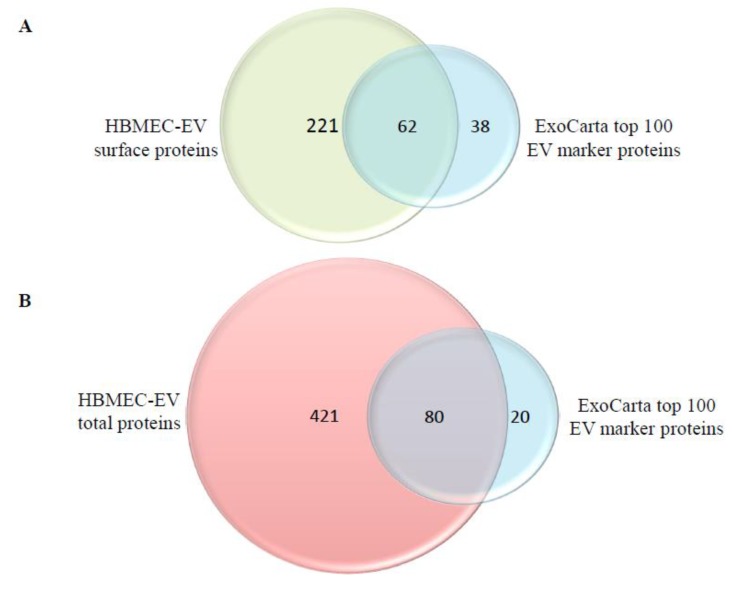

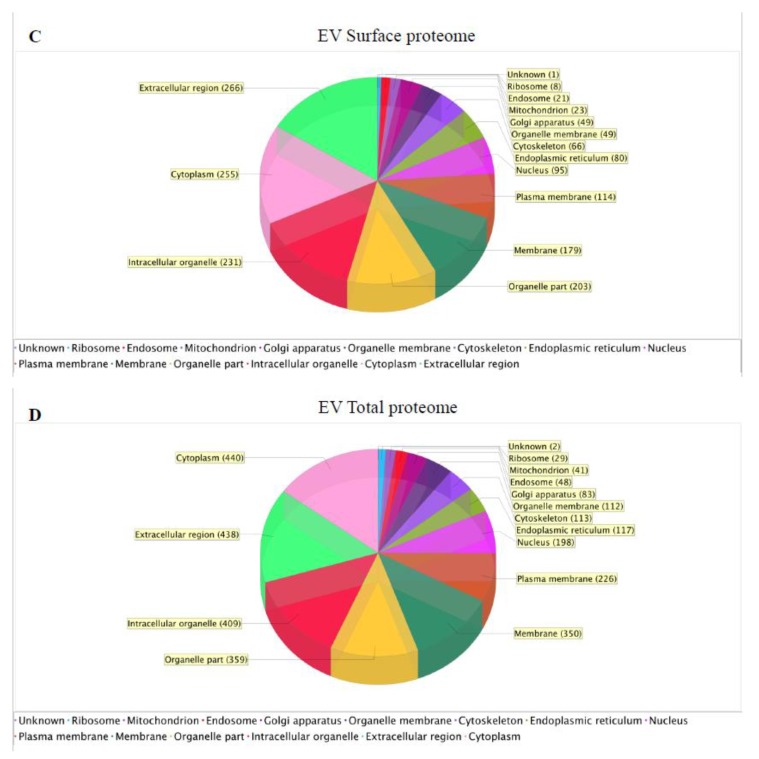
Extracellular vesicle (EV)-specific markers in the surface and total proteomes of human brain microvascular endothelial cells (HBMEC)-derived EVs. Venn diagram showing the overlap between the HBMEC-EV surface proteome (283 proteins) (**A**) or the HBMEC-EV total proteome (501 proteins) (**B**) and the top 100 EV marker proteins from ExoCarta. Cellular component enrichment of the identified surface (**C**) and total (**D**) EV proteomes. The identified EV proteins were enriched for cellular component using the Scaffold software.

At first, we examined whether proteins that are frequently identified in EVs/exosomes from various sources can be found in our isolated HBMEC-EVs. Based on the ExoCarta EV proteomics database from different human cell types that have been isolated using different approaches [19,20], we compiled the list of 100 marker proteins that are most often present on EVs (Table 1). The surface HBMEC-EV proteome, which contained a total of 283 identified proteins, included 62 of the top 100 ExoCarta EV markers (Figure 1A, Table 1). In addition, the total HBMEC-EV proteome, which contained 501 identified proteins, included 80 of such markers (Figure 1B, Table 1). These results demonstrate that our HBMEC-EV isolation was highly enriched with known EV markers. 

### 2.2. Cellular Component Enrichment of the Identified Surface and Total EV Proteins

Using the Scaffold software, we next evaluated the HBMEC-EV proteins according to their known cellular localization. This approach may indicate the parent cellular compartment origin of the identified HBMEC-EV proteins. The majority of the HBMEC-EV surface proteins were extracellular region proteins, followed by cytoplasmic, intracellular organelle, membrane, nuclear, endoplasmic reticulum, cytoskeleton, Golgi, mitochondrial, endosomal, ribosomal proteins, and one unknown protein (Figure 1C). For the total HBMEC-EV proteome, the majority of proteins were cytoplasmic and extracellular region proteins (Figure 1D). 

### 2.3. HIV and Aβ Exposure Results in Unique HBMEC-EV Proteome Signatures

**Figure 2:**
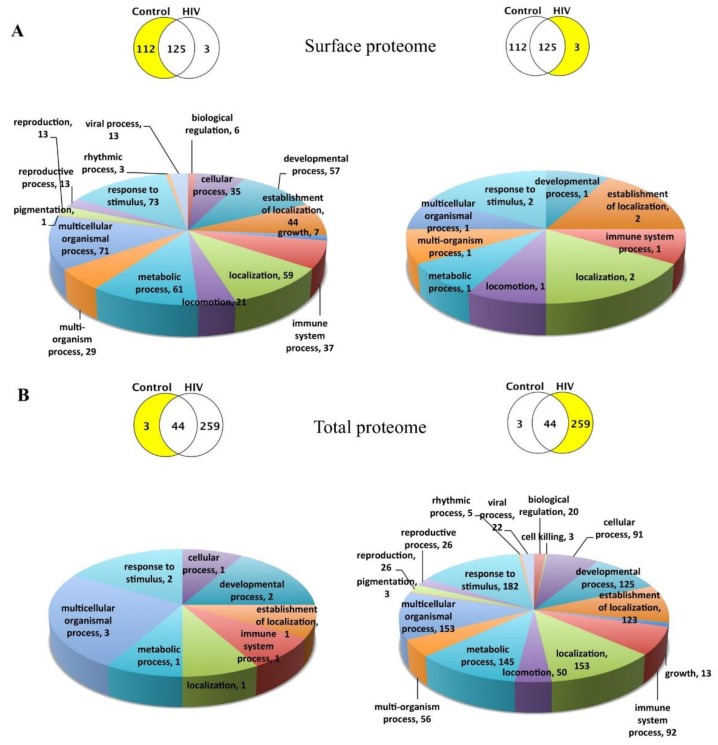

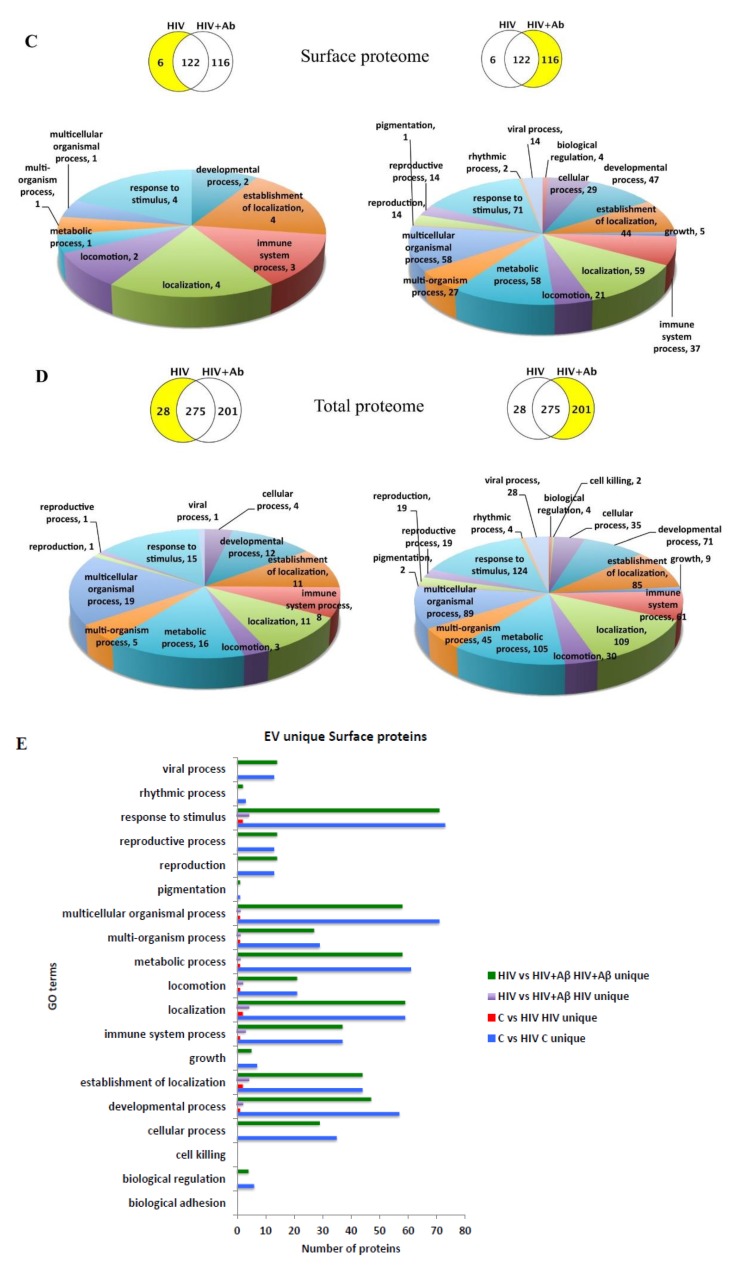

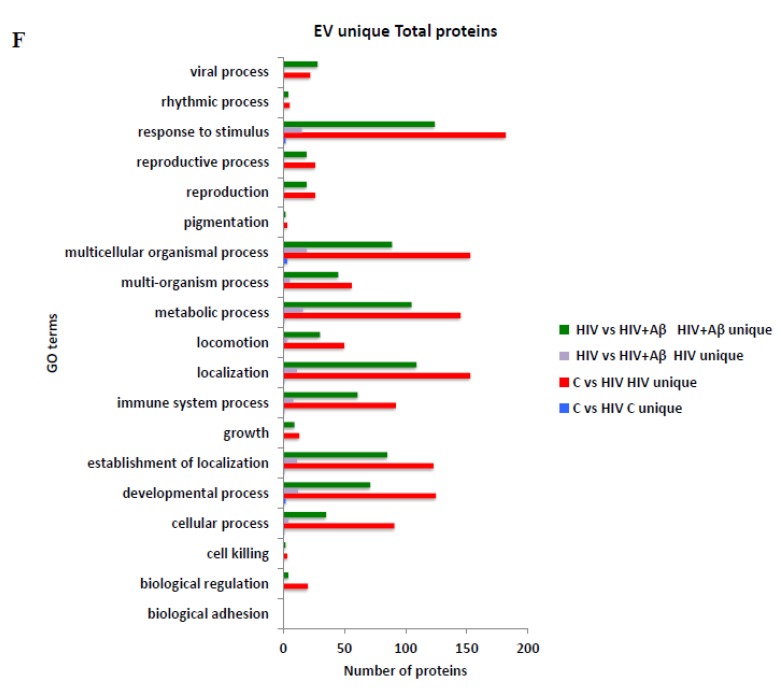
Enrichment for biological processes of the identified unique EV proteins. Scaffold software was used to enrich for the main biological processes for the identified unique EV proteins. The upper Venn diagrams show the compared groups with the number of their unique and shared proteins. The lower pie charts depict the enriched biological processes corresponding to the unique lists highlighted in yellow. The number of proteins in a particular biological process category is also provided. (**A**) Surface proteome, control vs. HIV. (**B**) Total proteome, control vs. HIV. (**C**) Surface proteome, HIV vs. HIV+ amyloid beta (Aβ). (**D**) Total proteome, HIV vs. HIV+Aβ. Combined graph for the biological processes in the EV unique surface (**E**) and total (**F**) proteomes. The number of unique proteins corresponding to the main biological processes in the different comparisons is illustrated on the graph.

We next focused on the unique proteins induced by the exposure to HIV and Aβ. Comparison of the control vs. HIV surface HBMEC-EV proteomes identified 112 unique proteins in the control and three unique proteins in the HIV group (Figure 2A). By contrast, a similar comparison for the total proteome identified only three unique proteins in the control and as many as 259 unique proteins in the HIV group (Figure 2B). Comparison of the surface proteome between the HIV vs. HIV+Aβ groups identified six unique proteins in the HIV group and 116 unique proteins in the HIV+Aβ group (Figure 2C). Finally, analysis of the total proteome revealed 28 unique proteins in the HIV group and 201 unique proteins in the HIV+Aβ group (Figure 2D). A list of these unique proteins is provided in Table 2 and Table 3 for the surface and total proteomes, respectively.

### 2.4. Functional Enrichment of the Unique HBMEC-EV Proteins

We next grouped these unique protein signatures into the biological process categories of the Scaffold software. Overall, 19 main categories were established, and the number of unique proteins mapping to these categories is illustrated in Figure 2, separately for the surface (A and C) and the total proteome (B and D). Note that individual proteins could map to more than one category; on the other hand, not all categories have been identified for all comparisons. This is consistent with the fact that selected group comparisons identified only a limited number of unique proteins that mapped to a limited number of categories. The number of unique proteins corresponding to the main biological process categories in the combined comparisons is illustrated on the bar graphs in Figure 2E for the surface proteome and Figure 2F for the total proteome. The majority of both surface and total unique proteins were mapped to “response to stimulus,” ”multicellular organismal process,” ”metabolic process,” and “localization” categories.

Next, we evaluated the unique proteins in the control vs. HIV and in the HIV vs. HIV+Aβ comparisons using STRING for functional enrichment in the biological processes and the Kyoto Encyclopedia of Genes and Genomes (KEGG) Pathways. In addition, we enriched these analyses for cellular components and PMID publications.

The results of these analyses for the EV surface proteome unique proteins in the control group in the control vs. HIV comparison are listed in Table 4 and Supplementary Table S1A. In addition, Supplementary Table S1B lists the enrichment for cellular components. The observed gene count (Obs), background gene count (Bgr), false discovery rate (FDR), and matched proteins are also included in these tables. The three unique proteins identified when comparing the surface proteome in the HIV group to the control group are dynein heavy chain 8, axonemal (DNAH8), titin (TTN), and immunoglobulin heavy constant gamma 2 (IGHG2). According to the description in the STRING or GeneCards database, DNAH8 is a force-generating protein of the respiratory cilia and is also involved in sperm motility. In addition, DNAH8 is highly expressed in prostate cancer [21]. Titin appears to be a key component of the vertebrate striated muscles [22]. IGHG2 may take part in antigen binding and the regulation of actin dynamics. It was linked to severe respiratory syncytial virus infection [23]. Overall, very limited or no data were found for the different enrichment analyses in STRING regarding these three proteins.

Next, we analyzed the EV surface proteome unique lists for the HIV vs. HIV+Aβ comparison in order to dissect the effect of exogenous EV-Aβ cargo in the context of HIV. In this analysis, six unique proteins were identified in the HIV group, namely, TTN, ninein (NIN), DNAH8, adenylyl cyclase-associated protein 1 (CAP1), actin-related protein 2/3 complex subunit 4 (ARPC4), and IGHG2. For these unique proteins, all enriched biological processes are shown in Table 5. No KEGG pathways were enriched; however, several PMID publications were found by textmining (Table 5). Cellular localization of these enriched proteins to only a few categories was found, namely, “cytoskeletal part” (ARPC4, CAP1, DNAH8, NIN, TTN), “actin cytoskeleton” (ARPC4, CAP1, TTN), “supramolecular fiber” (DNAH8, NIN, TTN), “microtubule” (DNAH8, NIN), “ciliary part” (DNAH8, NIN), and “cytoplasmic region” (CAP1, DNAH8). 

For the unique proteins in the HIV+Aβ group in this comparison, the enriched biological processes, KEGG pathways, and PMID publications are presented in Table 6 and Supplementary Table S2A. The enrichment for cellular components is included in Supplementary Table S2B.

Next, we analyzed the EV total proteome unique lists for the control vs. HIV comparison. For the unique proteins in the control group, no gene ontology (GO) terms were found for biological processes. Similarly, no KEGG Pathways were enriched, likely because only three unique proteins were identified in this group and comparison. The cellular localization of these proteins is presented in Supplementary Table S3. In addition, the first 10 PMID publications enriched are shown in Table 7. The total proteome revealed 259 unique proteins in the HIV group that mapped to a variety of GO terms for biological processes (Table 8 and Supplementary Table S4A). They were also enriched in several KEGG pathways (Table 8) and assigned to diverse cellular components, as listed in Supplementary Table S4B. Textmining resulted in an unbiased PubMed search with the 10 most significant publications listed in Table 8. 

Finally, we analyzed the list of the unique proteins present in the total HBMEC-EV proteome in the HIV and HIV+Aβ groups. The unique proteins in the HIV group in this comparison mapped to only one GO term for biological processes, namely, “cell envelope organization,” presented in Table 9. No KEGG pathways and no cellular components were enriched for this group. The first 10 textmined PMID citations are presented in Table 9. The unique proteins in the HIV+Aβ group were enriched to several biological processes, KEGG pathways, and PMID publications (Table 10 and Supplementary Table S5A). Supplementary Table S5B lists the enrichments for the cellular component in this group. 

### 2.5. Analysis of Unique Protein Interactions

We also explored in STRING whether these unique proteins have functional interactions among each other. The statistical background assumed for this enrichment analysis was the whole human genome. We filtered our search for established interactions only for the input proteins, for the highest confidence (over 0.900), and for a static map without the protein structures. In the obtained interaction maps, different nodes are connected with colored lines depending on the functional association type. The results imply that the identified proteins have more interactions among themselves than what would be expected for a random set of proteins of similar size, drawn from the genome. Such enrichments indicate that the proteins are, at least partially, biologically connected as a group and may contribute jointly to shared functions.

**Figure 3:**
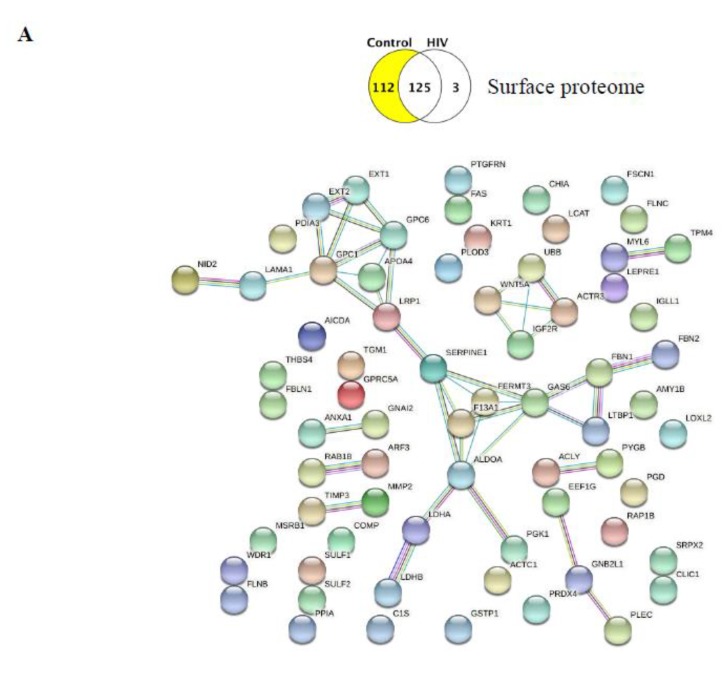

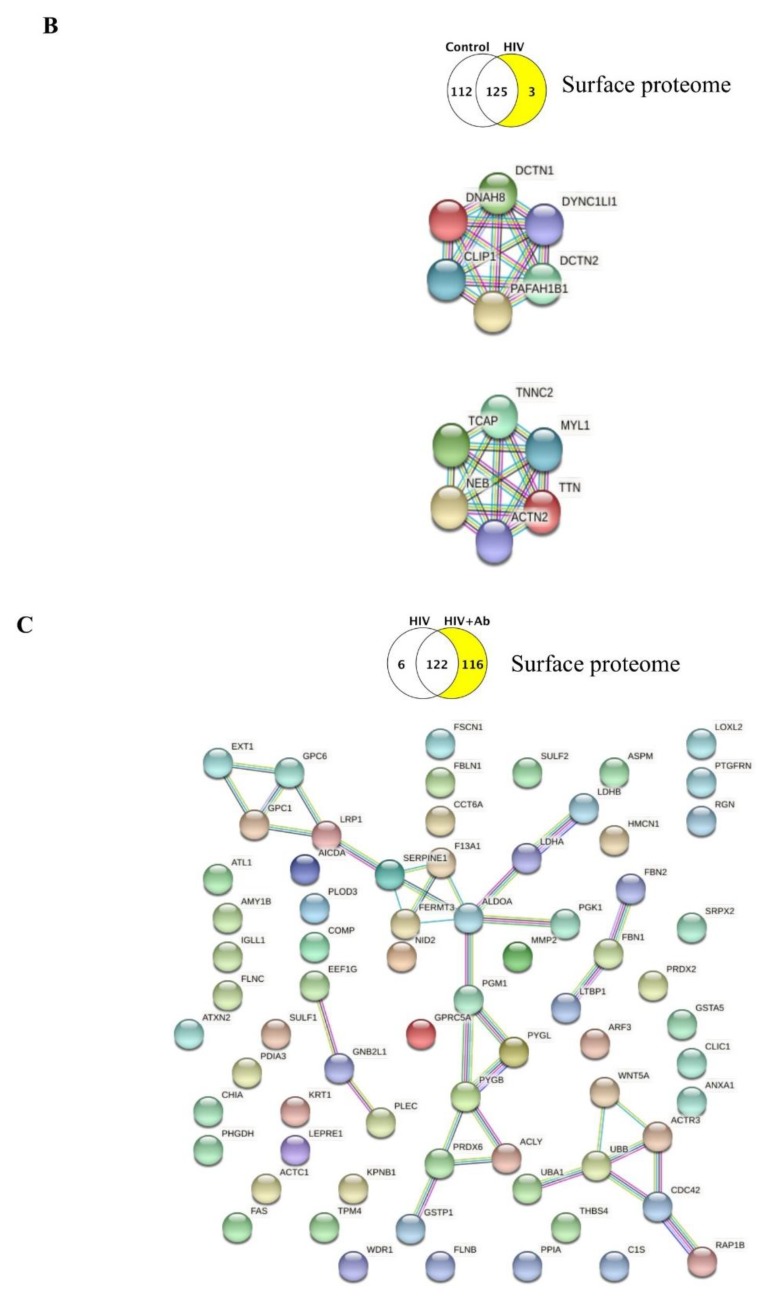
Protein–protein interactions between the identified unique proteins of the EV surface proteome. Venn diagrams illustrating the type of comparison and the number of identified unique proteins (highlighted). (**A**) Protein–protein interactions (PPI) (STRING) among the unique surface proteins in the control group. Only interactions with the highest confidence are shown with a minimum required interaction score of 0.900 (PPI enrichment p-value: 6.59 × 10^−7^; the network has significantly more interactions than expected). Known interactions: From curated databases (turquoise), experimentally determined (pink); predicted interactions: Gene neighborhood (green), gene fusions (red), gene co-occurrence (blue); other interactions: Textmining (light green), co-expression (black), protein homology (purple). (**B**) No interactions with highest confidence were identified in STRING among the three unique proteins identified in the HIV group. Predicted functional partners of dynein heavy chain 8, axonemal (DNAH8) (upper map) and titin (TTN) (lower map). Only the first shell of five interactions with the highest confidence is shown. Color code of the interaction lines as described in (**A**). (**C**) Protein–protein interactions among the unique proteins in the HIV+Aβ group. Only interactions with the highest confidence are shown (PPI enrichment p-value: 0.00158; the network has significantly more interactions than expected). Color code of the interaction lines as described in (**A**).

The interactions of the 112 unique surface proteins in the control group as compared to the HIV group are illustrated in Figure 3A. The HIV group in this comparison had only three unique surface proteins (DNAH8, TTN, and IGHG2). Being present on the EV surface, these proteins may be prone to interact with their potential functional partners beyond the EV surface. Therefore, we examined their possible interactions not only with each other but with other proteins as well. The STRING program identified predicted functional partners for DNAH8 and TTN, and the top five candidates that were predicted with the highest confidence, as well as their interacting networks, are illustrated in Figure 3B.

Next, we evaluated the unique surface protein list in the HIV vs. HIV+Aβ group. No protein–protein interactions were found for the six proteins uniquely expressed in the HIV group. By contrast, the HIV+Aβ unique surface proteins had several complex interactions, as illustrated in Figure 3C.

**Figure 4:**
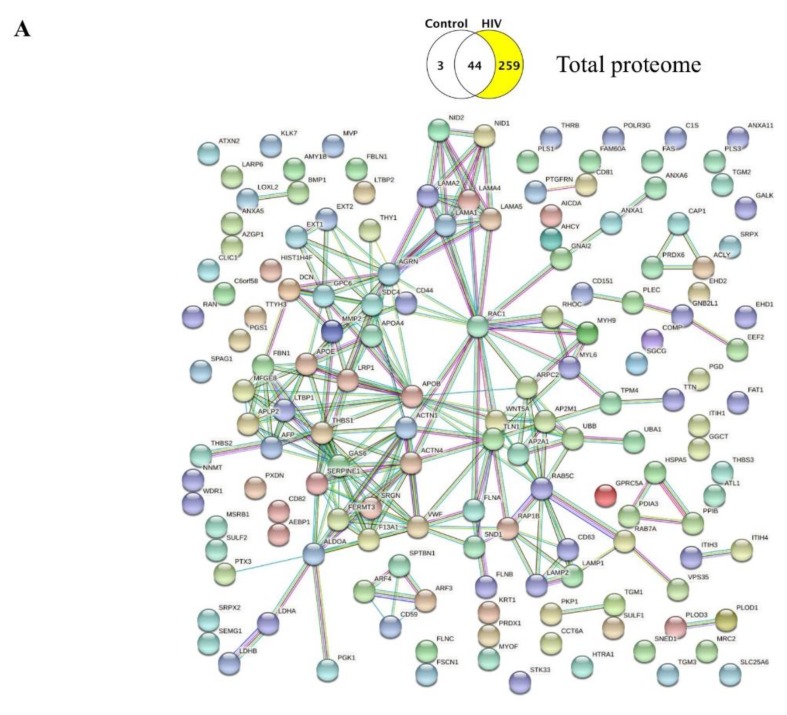

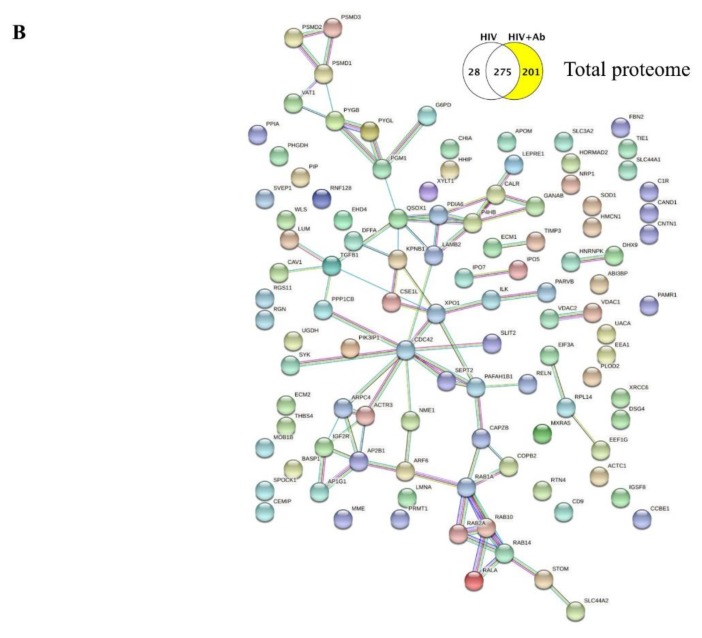
Protein–protein interactions in the identified unique proteins of the EV total proteome. Venn diagrams illustrating the type of comparison and the number of identified unique proteins (highlighted). (**A**) Protein–protein interactions among the unique proteins in the HIV group. Only interactions with the highest confidence are shown (PPI enrichment p-value: 1.0 × 10^−16^; the network has significantly more interactions than expected). (**B**) Protein–protein interactions among the unique proteins in the HIV+Aβ group. Only interactions with the highest confidence are shown (PPI enrichment p-value: 1.45 × 10^−7^; the network has significantly more interactions than expected). Color code of the interaction lines as described in Figure 3A.

Finally, we analyzed the interactions between the unique proteins present in the total HBMEC-EV proteome. No interactions were found in the control group as compared to the HIV group; however, the elaborate interaction map for the total unique proteins in the HIV group is presented in Figure 4A. For the HIV vs. HIV+Aβ comparison, the HIV group exhibited 28 unique proteins without any identified interactions. In contrast, the unique proteins in the HIV+Aβ group showed a complicated interaction network, as illustrated in Figure 4B.

## 3. Discussion

In the current study, we evaluated HBMEC-EV surface and total proteome changes evoked by HIV-1 alone and together with Aβ. We limited our analyses to the unique lists of proteins identified in the treatment groups; thus, we did not include the shared protein lists and the complex changes in the up- and down-regulated proteins. In addition, we specifically focused on the unique proteins in the control vs. HIV and in the HIV vs. HIV+Aβ group comparisons. The identified proteins were mapped to different gene ontology (GO) terms for biological processes, KEGG pathways, and Cell Components. We also explored the protein–protein interactions among the identified unique proteins.

Overall, the surface proteome control vs. HIV comparison indicated that the functions of the identified unique proteins ranged from diverse biological processes in the control (mainly “extracellular matrix organization,” “metabolic processes,” “vesicle-mediated transport,” “exocytosis”) and KEGG pathways (mainly “proteoglycans in cancer,” “focal adhesion,” “carbohydrate and cholesterol metabolism,” “HIF-1 signaling pathway”) to few or no distinct biological processes in the HIV group (Figure 2A and Table 4). The latter phenomenon was likely due to the limited number of proteins (namely, DNAH8, TTN, IGHG2) that were unique in the HIV-1 group when compared to the HBMEC-EV surface proteome of the controls. Nevertheless, we found several potential functional partners for DNAH8, such as platelet-activating factor acetylhydrolase IB subunit alpha (PAFAH1B1), dynactin subunit 1 (DCTN1), dynactin subunit 2 (DCTN2), CAP-Gly domain-containing linker protein 1 (CLIP1), and cytoplasmic dynein 1 light intermediate chain 1 (DYNC1LI1). Similarly, we identified several predicted functional partners for TTN, namely, nebulin (NEB), telethonin (TCAP), troponin C, skeletal muscle (TNNC2), myosin light chain 1/3, skeletal muscle isoform (MYL1), and alpha-actinin-2 (ACTN2) (Figure 3B). Thus, these few unique surface EV proteins in the HIV group may engage primarily with proteins of actin cytoskeleton/microtubule remodeling and vesicle-mediated transport.

The control EV proteome exhibited more than a hundred unique proteins; thus, it appears that after HIV-1 exposure of the parent cells, the EV surface proteome almost completely “blended” into the control proteome. This relative lack of surface HBMEC-EV protein signature in the HIV group is particularly striking in light of our previous findings where the exposure of HBMEC to HIV results in increased EV shedding [18] and the fact that EVs are involved in spreading HIV infection to the neighboring cells. However, it is possible that the localization of some proteins could alter from the EV surface to the vesicle lumen, resulting in a highly enriched total but not surface proteome. Indeed, comparison of the total proteome revealed a highly diverse number of 259 unique proteins in the HIV group as compared to the control that mapped to a variety of biological processes and KEGG pathways. The most prominent enrichment among the biological processes category was “vesicle-mediated transport,” followed by “extracellular structure organization.” In addition, mapping these unique proteins to “exocytosis” and “secretion by cell” categories points to processes that may be involved in EV release and EV transport (Figure 2B and Table 8). Likewise, the KEGG pathways were also diverse, from “focal adhesion” and “endothelial cell medium (ECM)-receptor interaction” to “proteoglycans in cancer,” different infections, “endocytosis,” “cholesterol metabolism,” and “glycolysis/gluconeogenesis” (Table 8). Thus, the total EV proteome in the HIV group, with a large number of unique proteins, may suggest that the rich, unique cargo is somewhat “hidden” within the EVs with a surface proteome that was barely altered. This notion is supported by the observations that the HIV group in the HIV versus HIV+Aβ group surface proteome comparison also exhibited only six unique proteins (Figure 2C). On the other hand, the relative lack of unique EV surface protein signatures may facilitate EV internalization and, thus, HIV transmission to other cells.

In addition to the effects of HIV-1, we explored the impact of Aβ on the HBMEC-EV proteome in the context of HIV-1. It was reported that increased brain Aβ induced profound proteome remodeling in multiple cell types, altering brain molecular pathways in an Alzheimer’s disease (AD) mouse model [24]. Another brain proteomic study using a different AD mouse model with amyloid and neurofibrillary tangle pathologies indicated age-dependent immune responses and synaptic dysfunctions. It was proposed that these changes were evoked by the advancing Aβ pathology in the brain [25], further demonstrating the importance of proteomic analyses in studies on the mechanisms of amyloid pathology.

Comparison of surface proteomes of EVs derived from HBMEC exposed to HIV alone vs. HIV+Aβ revealed profound changes, as demonstrated by 116 unique proteins in the HIV+Aβ group (Figure 2C). Aβ, acting on a HIV background, appeared to shift biological processes from mainly actin cytoskeleton organization (Table 5) to immune responses, extracellular matrix organization, and carbohydrate metabolic processes. In addition, enrichment of the “vesicle-mediated transport” and “exocytosis” also pointed to processes involved in EV release and EV transport (Figure 2C and Table 6). The KEGG pathways changed from a “blended” profile in the HIV group to a very diverse profile in the HIV+Aβ group, pointing mainly to the carbohydrate metabolic processes, “focal adhesion,” different infections, and signaling pathways as demonstrated by HIF-1, MAPK, and AGE-RAGE enrichment (Table 6). Regarding these signaling pathways, we have shown before the involvement of the RAGE pathway in the HIV-induced Aβ accumulation in HBMEC [26].

The HIV vs. HIV+Aβ comparison for the total proteome indicated substantial remodeling in the HIV+Aβ with 201 unique proteins as compared to 28 of such proteins in the HIV group. Consistent with HIV+Aβ-mediated EV release [18], the biological processes changed from “cell envelope organization” (Table 9) to mainly “vesicle-mediated transport,” “exocytosis,” and immune responses (Figure 2D and Table 10). The KEGG pathways also shifted to a diverse profile. “Endocytosis” was the most significant, followed by “focal adhesion” and “bacterial invasion of epithelial cells.” Several proteins were part of the carbohydrate metabolic pathways, such as the “pentose phosphate pathway,” “starch and sucrose metabolism,” and “proteoglycans in cancer” (Table 10).

Surprisingly, surface and total proteome analysis across different groups did not find any Aβ species in EVs, not even in samples that were isolated from Aβ-exposed HBMEC. This lack of Aβ identification could be related to technical issues, such as aggregation of Aβ, its insolubility, and possibly indigestibility by trypsin. The tryptic peptide used to quantify β-amyloid, LVFFAEDVGSNK, corresponding to amino acids 688–699, maps to all species of Aβ and full-length APP [27] and has been identified in the human CSF proteome [28]. In our study, no peptides mapping to the Aβ-generating region of APP were identified, even though APP was identified on the surface proteome. Similar obstacles were described in another proteomic study, in which Aβ was not identified in human AD brains. However, Aβ was detected by dot blot and ELISA from the same samples [29], supporting the notion that the lack of Aβ detection in the proteome was likely due to technical limitations.

Our previous studies demonstrated that treatment of HBMEC with Aβ could enrich EVs with this peptide, which can then be carried and delivered to different cells of the neurovascular unit [18,30]. In support of these findings, literature reports described Aβ as being present on the EV surface. For example, neuron-derived EVs accelerated Aβ fibril formation from monomeric Aβ, and this process was inhibited by cleavage of glycosphingolipid (GSL) glycans by endoglycoceramidase (EGCase) [31]. The same group also demonstrated that EV GSL-glycans were critical for Aβ binding in vitro and in vivo [15]. GSLs are found mainly in lipid rafts in the outer layer plasma membrane with their glycans facing outside; however, they are more abundant in EVs than in the parent cells [15]. Besides GSL, EVs were shown to bind Aβ through the prion protein (PrP) [14], a glycosylphosphatidylinositol-anchored protein in the outer leaflet of the neuron and neuron-derived EV membrane [32].

Some of the unique proteins identified in our HBMEC-derived EVs exhibit a substantial overlap with proteins detected by label-free proteomics in Aβ-enriched extracts from human AD brains [29], suggesting the relevance of EV proteins to Aβ pathology. The examples include ANXA5, FGB, LAMA5, and VIM found both in the total proteome of EVs in the HIV group and in Aβ-enriched extracts from human AD brains [29]. In addition, specific types of tubulins, such as TUBA1B and TUBB4B, were present, although they did not change in AD brains. Among the unique proteins in the HIV+Aβ group’s total proteome, FGG and HIST1H2BK, as well as tubulins TUBB and TUBB2A, were also enriched in extracts from AD brains [29]. In addition, HIST1H2BK has been one of the unique proteins in the EV total proteome from the Aβ group. In contrast, RNF213 was not identified in any of our EV samples, although it was unique to the AD brain samples and also found within the amyloid plaques [29]. One explanation for this phenomenon could be that RNF213 in the AD brain might not originate from brain endothelial cells.

Analysis for predicted significant functional interactions among the unique proteins produced several elaborate interaction maps (Figure 3 and Figure 4). It is striking to notice that several proteins on these maps act like “hubs” or centers by having a substantial number of connections to other proteins. Such “hubs” for the surface proteomes were SERPINE1 (PAI-1), GPC1, FERMT3 (Figure 3A), and ALDOA (Figure 3C). The most complex functional interaction maps were obtained for the total proteomes due to the high number of unique proteins. The identified “hubs” were RAC1, GAS6, SERPINE1, AGRN, APOB, and RAB5C (Figure 4A), as well as CDC42 and RAB1A (Figure 4B). Among these proteins, endothelial AGRN (agrin) was shown to be implicated in the brain Aβ pathology. For example, deletion of the *Agrn* gene from endothelial cells resulted in significantly increased Aβ levels in the mouse brain; however, overexpression of *Agrn* restored brain Aβ levels [33]. SERPINE1 (PAI-1) and GPC1 (glypican-1) may be additional important players in the Aβ pathology [34,35]. Indeed, GPC1, a heparan sulfate proteoglycan, localized mainly in detergent-insoluble, GSL-rich membrane domains, was shown to bind fibrillar Aβ in the human brain [36], further suggesting that protein “hubs” identified in the present study may be involved in EV-mediated Aβ pathology.

In summary, our results provide information, with an unprecedented resolution, on the brain endothelial surface and total EV proteome changes after HIV and Aβ exposure of the parent cells. The analyses identified protein–protein interaction networks, biological processes, pathways, and cellular localization. Overall, the obtained results factor for a better understanding of HBMEC-EV protein landscape changes induced by HIV and Aβ and their contribution to the HIV-associated Aβ pathology in the brain.

## 4. Materials and Methods

### 4.1. Cell Cultures

*Primary human brain microvascular endothelial cells (HBMEC)* used in the study were purchased from ScienCell Research laboratories (Carlsbad, CA, USA). HBMEC were isolated from human brain and cryopreserved at passage one. HBMEC were characterized by immunofluorescence with antibodies specific to vWF/Factor VIII and CD31 (PECAM). Cells were cultured on bovine plasma fibronectin (ScienCell)-coated dishes in endothelial cell medium (ECM). Specifically, 500 mL of basal ECM medium was supplemented with 25 mL of exosome-depleted fetal bovine serum (Exo-FBS; System Biosciences, Mountain View, CA, USA), 5 mL of endothelial cell growth supplement (ECGS, ScienCell), and 5 mL of penicillin/streptomycin solution (P/S, ScienCell). We initiated two separate cultures on 100 mm cell culture dishes to reduce the number of passages and subcultured the cells twice at the 1:4 ratio. This resulted in 32 confluent cultures, with the average cell number at the end of experiment of 9.065 × 10^7^ cells/dish. Sixteen confluent cultures were used for EV surface proteomics, and 16 for EV total proteomics. The treatment groups were: 1) Control exposed to vehicle, 2) Aβ alone, 3) HIV alone, 4) HIV plus Aβ, with four samples/group.

### 4.2. HIV Infection and Aβ Treatment

HIV-1 stock was generated using human embryonic kidney (HEK) 293T cells (ATCC, Manassas, VA, USA) transfected with pYK-JRCSF plasmid containing full-length proviral DNA. Throughout the study, HBMEC were exposed to HIV particles at the p24 level of 30 ng/mL as previously reported [37]. Treatment was terminated by removing the cell culture media for EV isolation.

Aβ (1–40) was purchased from Anaspec (San Jose, CA, USA) and dissolved in PBS. Freshly solubilized Aβ solutions without pre-aggregation were used for experiments as such a form of Aβ was demonstrated to induce proinflammatory reactions in isolated rat brain microvessels [38]. Cells were treated with Aβ (1–40) at the concentration of 100 nM for 48 h in complete medium. Although uptake of Aβ by the BBB occurs rapidly [39], we terminated the treatment at 48 h to allow more EV to be secreted into the culture medium. Confluent HBMEC were exposed to HIV-1 or/and Aβ (1–40) for 48 h.

### 4.3. EV Isolation

EV isolation was performed using ExoQuick-TC precipitation solution (System Biosciences) from conditioned culture media according to the manufacturer’s specifications. Briefly, 10 mL culture media from confluent HBMEC cultures was centrifuged at 3000 g for 15 min to remove cells and debris, and then mixed thoroughly with 2 mL of Exo-Quick precipitation solution and incubated overnight at 4 °C. The next day, samples were centrifuged at 1500 g for 30 min, and the supernatants were removed and centrifuged again at 1500 g for 5 min. The EV pellets were stored at –80 °C and used for proteomics analysis. Separate EV samples were prepared for EV surface and total proteomics.

### 4.4. Proteomics

*Sample Preparation.* Each sample was processed by SDS-PAGE using a 10% Bis Tris NuPage mini-gel (Invitrogen, Waltham, MA, USA) in the MES buffer system. The migration windows (1 cm lane) were excised and processed by in-gel digestion with trypsin using a ProGest robot (DigiLab) with the following protocol: The samples were washed with 25 mM ammonium bicarbonate followed by acetonitrile, reduced with 10 mM dithiothreitol at 60 °C, followed by alkylation with 50 mM iodoacetamide at room temperature, digested with trypsin (Promega, Madison, WI, USA) at 37 °C for 4 h, and quenched with formic acid. The supernatants were then analyzed directly without further processing.

*Mass Spectrometry.* Half of each digested sample was analyzed by nano LC-MS/MS with a Waters NanoAcquity HPLC system interfaced to a ThermoFisher Q Exactive. Peptides were loaded on a trapping column and eluted over a 75 μm analytical column at 350 nL/min; both columns were packed with Luna C18 resin (Phenomenex, Torrance, CA, USA). The mass spectrometer was operated in data-dependent mode, with the Orbitrap operating at 70,000 FWHM and 17,500 FWHM for MS and MS/MS respectively. The fifteen most abundant ions were selected for MS/MS.

*Data Processing.* Data were searched using Mascot (Matrix Science, London, UK; version 2.6.0) with the following parameters: Enzyme: Trypsin/P; Databases: SwissProt Human (concatenated forward and reverse plus common contaminants); fixed modifications: Carbamidomethyl (C); variable modifications: Acetyl (N-term), deamidation (N,Q), oxidation (M), Pyro-Glu (N-term Q); mass values: Monoisotopic; peptide mass tolerance: 10 ppm; fragment mass tolerance: 0.02 Da; max missed cleavages: 2. Mascot DAT files were parsed into Scaffold (Proteome Software, version Scaffold 4.8.7, Proteome Software Inc., Portland, OR, USA) for validation, filtering, and to create a non-redundant list per sample. Data were filtered using a 1% protein and peptide FDR and required at least two unique peptides per protein. Protein probabilities were assigned by the Protein Prophet algorithm [40]. Proteins that contained similar peptides and could not be differentiated based on MS/MS analysis alone were grouped to satisfy the principles of parsimony. Proteins were annotated with GO terms from NCBI (downloaded on Sep 6, 2018) [41]. The normalized spectral abundance factor (NSAF) calculation contains the conversion to the spectral abundance factor (SAF) and subsequent normalized spectral abundance factor (NSAF). This was based on the equation: NSAF = (SpC/MW)/Σ(SpC/MW)N, where SpC = spectral counts, MW = protein molecular weight in kDa, and N = total number of proteins. NSAF values can be used to approximate the relative abundance of proteins within a given sample and the relative abundance of a given protein between samples. The different treatment groups were compared using the *t*-test, and *p* < 0.05 was considered significant.

### 4.5. ExoCarta Database Search and Functional Enrichment Analysis

The list of the top 100 proteins most often identified in EVs was composed based on the ExoCarta EV proteomics database from different human cell types [19]. Enrichment in molecular functions of the identified EV proteins was analyzed using the Scaffold Proteome Software and STRING [42]. A gene ontology analysis study was carried out with the proteomic profiles obtained to identify overrepresentation profiles. Gene ontology was investigated at the levels of the biological process, KEGG pathways, and cell component. Textmining in STRING provided the most relevant publications for a particular enrichment. Kyoto Encyclopedia of Genes and Genomes (KEGG) established pathway maps representing molecular interactions, reactions, and relation networks for Metabolism, Genetic Information Processing, Environmental Information Processing, Cellular Processes, Organismal Systems, Human Diseases and Drug Development. KEGG PATHWAY is the reference database for pathway mapping in KEGG Mapper.

## Figures and Tables

**Table 1:** List of the top 100 ExoCarta proteins present in the brain endothelial extracellular vesicle (EV) surface (S) and total (T) proteome. Bold, top 100 ExoCarta proteins present in S or T; bold and red, proteins present in both S and T.

	Gene Symbol	Detected in S	Detected in T
1	**CD9**	−	+
2	**HSPA8**	+	+
3	**PDCD6IP**	+	+
4	**GAPDH**	+	+
5	**ACTB**	+	+
6	**ANXA2**	+	+
7	**CD63**	−	+
8	**SDCBP**	+	+
9	**ENO1**	+	+
10	**HSP90AA1**	+	+
11	**TSG101**	−	+
12	**PKM**	+	+
13	**LDHA**	+	+
14	**EEF1A1**	+	+
15	**YWHAZ**	+	+
16	**PGK1**	+	+
17	**EEF2**	+	+
18	**ALDOA**	+	+
19	**HSP90AB1**	+	+
20	**ANXA5**	+	+
21	**FASN**	+	+
22	**YWHAE**	+	+
23	**CLTC**	+	+
24	**CD81**	−	+
25	**ALB**	+	+
26	**VCP**	+	+
27	**TPI1**	+	+
28	**PPIA**	+	+
29	**MSN**	+	+
30	**CFL1**	+	+
31	**PRDX1**	+	+
32	**PFN1**	+	+
33	**RAP1B**	+	+
34	**ITGB1**	+	+
35	**HSPA5**	+	+
36	**SLC3A2**	−	+
37	**HIST1H4A**	+	+
38	GNB2	−	−
39	**ATP1A1**	−	+
40	**YWHAQ**	+	+
41	FLOT1	−	−
42	**FLNA**	+	+
43	**CLIC1**	+	+
44	**CDC42**	+	+
45	**CCT2**	+	+
46	**A2M**	+	+
47	**YWHAG**	+	+
48	**TUBA1B**	+	+
49	**RAC1**	−	+
50	**LGALS3BP**	+	+
51	**HSPA1A**	+	+
52	**GNAI2**	+	+
53	**ANXA1**	+	+
54	RHOA	−	−
55	**MFGE8**	−	+
56	**PRDX2**	+	−
57	**GDI2**	+	+
58	**EHD4**	−	+
59	**ACTN4**	+	+
60	YWHAB	−	−
61	**RAB7A**	−	+
62	**LDHB**	+	+
63	GNAS	−	−
64	TFRC	−	−
65	**RAB5C**	−	+
66	ARF1	−	−
67	**ANXA6**	+	+
68	**ANXA11**	−	+
69	ACTG1	−	−
70	**KPNB1**	+	+
71	**EZR**	−	+
72	ANXA4	−	−
73	**ACLY**	+	+
74	TUBA1C	−	−
75	**RAB14**	−	+
76	HIST2H4A	−	−
77	**GNB1**	+	+
78	**UBA1**	+	+
79	**THBS1**	+	+
80	**RAN**	+	+
81	RAB5A	−	−
82	**PTGFRN**	+	+
83	**CCT5**	+	+
84	**CCT3**	−	+
85	**BSG**	−	+
86	**AHCY**	+	+
87	RAB5B	−	−
88	**RAB1A**	−	+
89	**LAMP2**	−	+
90	ITGA6	−	−
91	HIST1H4B	−	−
92	**GSN**	+	+
93	**FN1**	+	+
94	**YWHAH**	−	+
95	**TUBA1A**	+	−
96	TKT	−	−
97	**TCP1**	+	+
98	**STOM**	−	+
99	SLC16A1	−	−
100	RAB8A	−	−

**Table 2:** List of the unique proteins in the EV surface proteome.

Control vs. HIV				HIV vs. HIV+Aβ			
Control Unique			HIV Unique	HIV Unique	HIV+Aβ Unique		
1433E	GPC6	TGM1	TITIN DYH8 IGHG2	TITIN NIN DYH8 CAP1 ARPC4 IGHG2	1433E	GPC1	TCPA
1433G	GSTP1	TIG1			1433G	GPC6	TCPB
1433T	IGL1	TIMP3			1A34	GSTA5	TCPE
1A34	ITA3	TPM4			1B15	GSTP1	TCPH
5NTD	ITAV	TRFE			5NTD	HMCN1	TCPZ
6PGD	ITB1	TSP4			ACLY	IGL1	TIG1
ACLY	K2C6B	UBB			ACTC	IMB1	TPM4
ACTC	LAMA1	UGPA			ADA10	ITA3	TRFE
ALDOA	LCAT	URP2			AL9A1	ITA5	TSP4
AMPN	LDHA	VINC			ALDOA	ITAV	UBA1
AMY1	LDHB	WDR1			AMY1	ITB1	UBB
ANXA1	LOXL2	WNT5A			ANXA1	LDHA	UGPA
APOA4	LRC17				ARF3	LDHB	URP2
ARF3	LRP1				ARP2	LOXL2	VINC
ARGI1	LTBP1				ARP3	LRC17	WDR1
ARP2	MIME				ASPM	LRP1	WNT5A
ARP3	MMP2				ATL1	LTBP1	
ATS13	MPRI				ATX2	MIME	
C1S	MYL6				B4GA1	MMP2	
CASPE	NID2				C1S	NID2	
CCD80	P3H1				CAZA1	P3H1	
CFAH	PAI1				CCD80	PAI1	
CHIA	PCOC1				CDC42	PDC6I	
CLIC1	PDC6I				CHIA	PDIA3	
CO4A2	PDIA3				CHSS2	PGK1	
CO5A2	PGK1				CISY	PGM1	
CO7	PLEC				CLIC1	PLEC	
CO7A1	PLOD3				CLUS	PLOD3	
COBA1	PPIA				CO4A2	PPIA	
COF1	PRDX4				CO5A2	PRDX2	
COFA1	PYGB				CO7A1	PRDX6	
COMP	RAB1B				COBA1	PUR6	
EF1G	RACK1				COF1	PYGB	
ENOB	RAP1B				COFA1	PYGL	
EXT1	RLA0				COMP	RACK1	
EXT2	RS16				EF1G	RAP1B	
F13A	S10A9				EXT1	RGN	
FA11	SDCB1				F13A	RIMB1	
FAS	SEPR				FA11	RL12	
FBLN1	SPB12				FAS	S10A9	
FBN1	SPR1B				FBLN1	SDCB1	
FBN2	SPR2E				FBN1	SERA	
FLNB	SRCRL				FBN2	SERPH	
FLNC	SRPX2				FLNB	SPR2E	
FPRP	SULF1				FLNC	SRCRL	
FRIH	SULF2				FPRP	SRPX2	
FSCN1	SYTC				FRIH	SULF1	
GAS6	TAGL2				FRIL	SULF2	
GNAI2	TBA1A				FSCN1	SYTC	
GPC1	TCPD				GDIB	TAGL2	

**Table 3:** List of the unique proteins in the EV total proteome.

Control vs. HIV						
Control Unique	HIV Unique					
ACTC MYH1 TAU	1433T	CD81	GDIB	MVP	S10AB	URP2
	1433Z	CD82	GELS	MYH9	SAHH	VIME
	1A24	CLH1	GGCT	MYL6	SCRB2	VINC
	5NTD	CLIC1	GNAI2	MYOF	SDC4	VPS35
	6PGD	CO1A2	GPC6	NID1	SDCB1	VTNC
	A4	CO3A1	GRP78	NID2	SEPR	VWF
	ACLY	CO4A1	GSLG1	NNMT	SERPH	WDR1
	ACTN1	CO4A2	GTR1	OLFL3	SND1	WNT5A
	ACTN4	CO5	H31	PAI1	SNED1	ZA2G
	ADA10	CO5A1	H4	PDC6I	SPTB2	
	AEBP1	CO5A2	HEP2	PDIA3	SPTN1	
	AGRIN	CO6A2	HS90A	PFKAP	SRCRL	
	AHNK	CO7A1	HS90B	PGK1	SRGN	
	ALDOA	CO9	HSP7C	PGS1	SRPX	
	AMPN	COEA1	HTRA1	PGS2	SRPX2	
	AMY1	COFA1	IF4A1	PKP1	SULF1	
	ANT3	COIA1	IGHA1	PLEC	SULF2	
	ANX11	COMP	ITA3	PLMN	SYDC	
	ANXA1	CYTA	ITA4	PLOD1	SYTC	
	ANXA5	DPYL2	ITA5	PLOD3	TAGL2	
	ANXA6	DYHC1	ITAV	PLS1	TBA1B	
	AP2A1	EF1A1	ITB1	PLS3	TBB4B	
	AP2M1	EF2	ITIH1	PPIB	TCPA	
	APLP2	EHD1	ITIH3	PRC2A	TCPB	
	APOA4	EHD2	ITIH4	PRDX1	TCPD	
	APOB	EMIL1	KLK7	PRDX6	TCPH	
	APOE	ENOA	KPYM	PROF1	TCPQ	
	ARF3	ENPL	LAMA1	PSB5	TCPZ	
	ARF4	EXT1	LAMA2	PTX3	TENA	
	ARGI1	EXT2	LAMA4	PXDN	TERA	
	ARP2	F13A	LAMA5	RAB5C	TGM1	
	ARPC2	FA5	LAMP1	RAB7A	TGM2	
	AT1A1	FAS	LAMP2	RAC1	TGM3	
	ATL1	FAT1	LDHA	RACK1	THBG	
	ATS12	FBLN1	LDHB	RAN	THRB	
	ATS13	FBN1	LEG1	RAP1B	THY1	
	ATX2	FBX50	LORI	RB11A	TIG1	
	B4GA1	FETA	LOXL2	RHOC	TITIN	
	BGH3	FIBB	LRC17	RIMB1	TLN1	
	BMP1	FILA	LRP1	RL10A	TPIS	
	C1QT3	FLNA	LTBP1	RL12	TPM4	
	C1S	FLNB	LTBP2	RL13A	TRFE	
	CAP1	FLNC	LYSC	RL27	TRFL	
	CASPE	FPRP	MAMC2	RL6	TSN14	
	CATA	FSCN1	MARCS	RS16	TSP 1	
	CCD80	G3P	MFGM	RS3	TSP 2	
	CD151	GALK1	MMP2	RS4X	TSP 3	
	CD44	GAS6	MOES	RS8	TTYH3	
	CD59	GBB1	MOT4	RSSA	UBA1	
	CD63	GBG12	MRC2	S10A9	UBB	
**HIV vs. HIV+Aβ**						
**HIV unique**	**HIV+Aβ unique**					
AHNK	1433E	CO8B	MIME	RL3		
ARGI1	1433F	COF1	MOB1B	RL7		
ATX2	1433G	COPB2	MPRI	RL7A		
B4GA1	1B40	COR1A	MRP	RLA0		
CASPE	2AAA	COR1C	MXRA5	RS11		
CATA	4F2	CTL1	MYH16	RS18		
CYTA	ACTC	CTL2	NDKA	RS2		
FBX50	AK1A1	CTND1	NEP	RS20		
FILA	AL9A1	CYFP1	NIBL1	RS25		
GGCT	ALS	DHX9	NOTC3	RS3A		
HORN	ANGL2	DSG4	NRP1	RS9		
IGHA1	ANGL4	DX39B	OLM2B	RTN4		
K1C13	ANM1	ECM1	P3H1	RUVB1		
KLK7	ANR31	ECM2	PAMR1	SC23A		
LORI	AP1G1	EEA1	PARVB	SCUB3		
LYSC	AP2B1	EF1G	PCOC1	SEM3C		
MYOF	APOM	EGLN	PDIA1	SEP11		
PLS1	ARF6	EHD4	PDIA6	SEPT2		
PRC2A	ARP3	EIF3A	PDLI5	SERA		
RIMB1	ARPC4	EZRI	PGFRB	SLIT2		
RL27	ASSY	FA10	PGM1	STOM		
S10A9	AT1B3	FA11	PIP	SVEP1		
SNED1	ATPA	FBN2	PLOD2	SYFB		
SPB12	ATPB	FIBG	PP1B	SYHC		
TGM1	ATS7	FRIH	PPIA	SYK		
TGM3	B4GT5	G6PD	PRS23	SYRC		
TITIN	BASI	G6PI	PRS8	SYSC		
ZA2G	BASP1	GANAB	PSA3	TARSH		
	BGAL	H13	PSA6	TBB2A		
	C1R	H2A1	PSD11	TBB5		
	C1TC	H2B1K	PSD12	TCPE		
	CAD23	HGFL	PSD13	TCPG		
	CALR	HHIP	PSMD1	TGFB1		
	CAND1	HMCN1	PSMD2	TICN1		
	CAPZB	HNRPK	PSMD3	TIE1		
	CAV1	IGSF8	PUR6	TIMP3		
	CAZA1	ILK	PYGB	TS101		
	CBPN	IMB1	PYGL	TSN6		
	CCBE1	IPO5	QSOX1	TSN9		
	CD9	IPO7	RAB10	TSP4		
	CDC42	IQGA1	RAB14	UACA		
	CEMIP	KCRM	RAB1A	UGDH		
	CFAH	KR101	RAB2A	VAT1		
	CHIA	KR111	RALA	VDAC1		
	CHSS2	KRA11	RELN	VDAC2		
	CISY	LAMB2	RGN	VGFR1		
	CLUS	LIS1	RL14	XPO1		
	CNTN1	LMNA	RL18	XPO2		
	CO7	LRC15	RL18A	XPP1		
	CO8A	LUM	RL22	XRCC6		
				XYLT1		

**Table 4:** Biological processes, KEGG pathways, and PMIDs for the EV surface unique proteins in the control group for the control vs. HIV comparison.

**Gene Ontology (GO) Terms for Biological Processes** **10 Most Significant Results per FDR (for all GO Terms, See Supplemental Table S1A)**					
**Term description**		**Obs**	**Bgr**	**FDR**	**Matching proteins in the network**
Extracellular structure organization		16	339	2.01 × 10^−10^	APOA4,COMP,FBLN1,FBN1,FBN2,GAS6,LAMA1,LCAT,LOXL2,MMP2,NID2,PLOD3,PRDX4,SERPINE1,SULF1,SULF2
Extracellular matrix organization		14	296	4.25 × 10^−9^	COMP,FBLN1,FBN1,FBN2,GAS6,LAMA1,LOXL2,MMP2,NID2,PLOD3,PRDX4,SERPINE1,SULF1,SULF2
Organonitrogen compound metabolic process		42	5281	3.69 × 10 ^-6^	ACLY,AICDA,ALDOA,ANXA1,APOA4,C1S,CHIA,EEF1G,EXT1,EXT2,F13A1,FBLN1,FBN1,GAS6,GNB2L1,GPC1,GPC6,GSTP1,IGF2R,IGLL1,KRT1,LCAT,LDHA,LDHB,LEPRE1,LOXL2,LRP1,LTBP1,MMP2,MSRB1,PDIA3,PGD,PGK1,PLOD3,PPIA,PRDX4,RAB1B,SULF1,SULF2,TGM1,UBB,WNT5A
Immune response		22	1560	6.48 × 10^−6^	ACLY,ACTR3,AICDA,ALDOA,ANXA1,APOA4,C1S,CHIA,FAS,FLNB,GAS6,GSTP1,IGF2R,IGLL1,KRT1,LRP1,MSRB1,PPIA,PRDX4,PYGB,RAP1B,WNT5A
Vesicle-mediated transport		23	1699	6.48 × 10^−6^	ACLY,ACTR3,ALDOA,ANXA1,ARF3,F13A1,FERMT3,GAS6,GSTP1,IGF2R,IGLL1,KRT1,LOXL2,LRP1,PPIA,PRDX4,PYGB,RAB1B,RAP1B,SERPINE1,TIMP3,UBB,WDR1
Regulated exocytosis		15	691	6.48 × 10^−6^	ACLY,ALDOA,F13A1,FERMT3,GAS6,GSTP1,IGF2R,KRT1,PPIA,PRDX4,PYGB,RAP1B,SERPINE1,TIMP3,WDR1
Positive regulation of biological process		42	5459	6.48 × 10^−6^	ACLY,ACTC1,ACTR3,AICDA,ANXA1,APOA4,C1S,CHIA,CLIC1,FAS,FBLN1,FBN1,FBN2,FERMT3,FSCN1,GAS6,GNAI2,GNB2L1,GPC1,GSTP1,IGF2R,IGLL1,KRT1,LDHA,LEPRE1,LOXL2,LRP1,MMP2,PDIA3,PPIA,RAB1B,RAP1B,SERPINE1,SRPX2,SULF1,SULF2,TGM1,THBS4,TIMP3,UBB,WDR1,WNT5A
Anatomical structure development		40	5085	6.48 × 10^−6^	ACTC1,AICDA,ANXA1,APOA4,COMP,EXT1,EXT2,FAS,FBLN1,FBN1,FBN2,FERMT3,FLNB,FLNC,FSCN1,GAS6,GNB2L1,GPC1,GSTP1,IGF2R,KRT1,LDHA,LEPRE1,LOXL2,LRP1,LTBP1,MMP2,MYL6,PGK1,PLOD3,PRDX4,RAP1B,SERPINE1,SRPX2,SULF1,SULF2,TGM1,UBB,WDR1,WNT5A
Response to stimulus		51	7824	6.48 × 10^−6^	ACLY,ACTC1,ACTR3,AICDA,ALDOA,ANXA1,APOA4,C1S,CHIA,CLIC1,EEF1G,EXT1,EXT2,F13A1,FAS,FBLN1,FBN1,FERMT3,FLNB,FSCN1,GAS6,GNAI2,GNB2L1,GPC1,GPC6,GPRC5A,GSTP1,IGF2R,IGLL1,KRT1,LAMA1,LDHA,LOXL2,LRP1,LTBP1,MMP2,MSRB1,PDIA3,PGK1,PLOD3,PPIA,PRDX4,PYGB,RAP1B,SERPINE1,SULF1,SULF2,THBS4,TIMP3,UBB,WNT5A
Positive regulation of cellular process		39	4898	7.40 × 10^−6^	ACLY,ACTR3,AICDA,ANXA1,APOA4,CHIA,CLIC1,FAS,FBLN1,FBN1,FBN2,FERMT3,FSCN1,GAS6,GNAI2,GNB2L1,GPC1,GSTP1,IGF2R,IGLL1,LDHA,LEPRE1,LOXL2,LRP1,MMP2,PDIA3,PPIA,RAB1B,RAP1B,SERPINE1,SRPX2,SULF1,SULF2,TGM1,THBS4,TIMP3,UBB,WDR1,WNT5A
**KEGG Pathways**					
**Term Description**		**Obs**	**Bgr**	**FDR**	**Matching Proteins in the Network**
Proteoglycans in cancer		7	195	0.00093	FAS,FLNB,FLNC,GPC1,MMP2,TIMP3,WNT5A
Focal adhesion		6	197	0.0053	COMP,FLNB,FLNC,LAMA1,RAP1B,THBS4
Glycolysis / Gluconeogenesis		4	68	0.0054	ALDOA,LDHA,LDHB,PGK1
HIF-1 signaling pathway		4	98	0.0155	ALDOA,LDHA,PGK1,SERPINE1
Cholesterol metabolism		3	48	0.0195	APOA4,LCAT,LRP1
Malaria		3	47	0.0195	COMP,LRP1,THBS4
**10 Most Significant PMID Publications per FDR**					
**Term ID**	**Term Description**	**Obs**	**Bgr**	**FDR**	**Matching Proteins in the Network**
PMID:21654676	(2011) D-glucuronyl C5-epimerase suppresses small-cell lung cancer cell proliferation in vitro and tumour growth in vivo.	8	62	1.79 × 10^−5^	EXT1,EXT2,FAS,GPC1,GPC6,MMP2,SERPINE1,TIMP3
PMID:22393382	(2012) In vitro phenotypic, genomic and proteomic characterization of a cytokine-resistant murine Beta-TC3 cell line.	7	42	2.32 × 10^−5^	ALDOA,FAS,GSTP1,LDHA,LDHB,PDIA3,PRDX4
PMID:25829250	(2015) Insights into the key roles of proteoglycans in breast cancer biology and translational medicine.	10	156	2.32 × 10^−5^	EXT1,FBLN1,FBN1,GPC1,GPC6,MMP2,SULF1,SULF2,TIMP3,WNT5A
PMID:26779482	(2015) The Extracellular Matrix in Bronchopulmonary Dysplasia: Target and Source.	7	41	2.32 × 10^−5^	FBLN1,FBN1,FBN2,LOXL2,LTBP1,PLOD3,SULF2
PMID:23143224	(2013) The biology of the extracellular matrix: Novel insights.	6	28	5.53 × 10^−5^	COMP,FBN1,FBN2,LTBP1,MMP2,TIMP3
PMID:24223867	(2013) Lactate-modulated induction of THBS-1 activates transforming growth factor (TGF)-beta2 and migration of glioma cells in vitro.	6	31	7.90 × 10^−5^	COMP,LDHA,LDHB,MMP2,SERPINE1,THBS4
PMID:26076122	(2015) Interactions of signaling proteins, growth factors and other proteins with heparan sulfate: Mechanisms and mysteries.	6	31	7.90 × 10^−5^	EXT1,EXT2,GPC1,GPC6,SULF1,SULF2
PMID:20236620	(2010) Unraveling the mechanism of elastic fiber assembly: The roles of short fibulins.	6	33	8.27 × 10^−5^	FBLN1,FBN1,FBN2,LOXL2,LTBP1,TIMP3
PMID:20140087	(2010) Comprehensive identification and modified-site mapping of S-nitrosylated targets in prostate epithelial cells.	8	103	8.31 × 10^−5^	ALDOA,ANXA1,CLIC1,FLNB,FLNC,PDIA3,PGK1,PLEC
PMID:27513329	(2016) Differential Expression Pattern of THBS1 and THBS2 in Lung Cancer: Clinical Outcome and a Systematic-Analysis of Microarray Databases.	7	65	8.31 × 10^−5^	COMP,FBLN1,FBN1,MMP2,NID2,SULF1,THBS4

**Table 5:** Biological processes and PMIDs for the EV surface unique proteins in the HIV group for the HIV vs. HIV+Aβ comparison.

**Gene Ontology (GO) Terms for Biological Processes**					
**Term Description**		**Obs**	**Bgr**	**FDR**	**Matching Proteins in the Network**
Cytoskeleton organization		5	953	8.35 × 10^−5^	ARPC4,CAP1,DNAH8,NIN,TTN
Supramolecular fiber organization		4	383	0.00011	ARPC4,CAP1,NIN,TTN
Actin filament organization		3	200	0.0011	ARPC4,CAP1,TTN
Cellular protein-containing complex assembly		4	832	0.0012	ARPC4,DNAH8,NIN,TTN
Actin polymerization or depolymerization		2	43	0.0031	ARPC4,CAP1
Protein polymerization		2	83	0.0058	ARPC4,NIN
Localization		5	5233	0.0296	ARPC4,CAP1,DNAH8,NIN,TTN
**PMID Publications**					
**Term ID**	**Term Description**	**Obs**	**Bgr**	**FDR**	**Matching Proteins in the Network**
PMID:21050039	(2010) Titin A-band-specific monoclonal antibody Tit1 5H1.1. Cellular Titin as a centriolar protein in non-muscle cells.	2	2	0.0016	NIN,TTN
PMID:22985877	(2012) Epitope of titin A-band-specific monoclonal antibody Tit1 5 H1.1 is highly conserved in several Fn3 domains of the titin molecule. Centriole staining in human, mouse and zebrafish cells.	2	6	0.0037	NIN,TTN
PMID:26655833	(2016) The centrosome is an actin-organizing centre.	2	12	0.0081	ARPC4,NIN
PMID:27094867	(2016) Mutations in human C2CD3 cause skeletal dysplasia and provide new insights into phenotypic and cellular consequences of altered C2CD3 function.	2	27	0.027	NIN,TTN
PMID:29255378	(2017) The human, F-actin-based cytoskeleton as a mutagen sensor.	2	35	0.0353	DNAH8,TTN

**Table 6:** Biological processes, KEGG pathways, and PMIDs for the EV surface unique proteins in the HIV+Aβ group for the HIV vs. HIV+Aβ comparison.

**Gene ontology (GO) Terms for Biological Processes** **10 Most Significant Results per FDR (for All GO Terms, See Supplementary Table S2A)**					
**Term Description**		**Obs**	**Bgr**	**FDR**	**Matching Proteins in the Network**
Immune effector process		18	927	9.47 × 10^−6^	ACLY,ACTR3,AICDA,ALDOA,C1S,CDC42,GSTP,IGLL1,KPNB1,KRT1,LRP1,PGM1,PPIA,PRDX6,PYGB,PYGL,RAP1B,WDR1
Leukocyte-mediated immunity		15	632	9.47 × 10^−6^	ACLY,AICDA,ALDOA,C1S,GSTP1,IGLL1,KPNB1,KRT1,PGM1,PPIA,PRDX6,PYGB,PYGL,RAP1B,WDR1
Vesicle-mediated transport		23	1699	9.70 × 10^−6^	ACLY,ACTR3,ALDOA,ANXA1,ARF3,CDC42,F13A1,FERMT3,GSTP1,IGLL1,KPNB1,KRT1,LOXL2,LRP1,PGM1,PPIA,PRDX6,PYGB,PYGL,RAP1B,SERPINE1,UBB,WDR1
Extracellular matrix organization		11	296	9.70 × 10^−6^	COMP,FBLN1,FBN1,FBN2,LOXL2,MMP2,NID2,PLOD3,SERPINE1,SULF1,SULF2
Regulated exocytosis		15	691	1.15 × 10^−5^	ACLY,ALDOA,F13A1,FERMT3,GSTP1,KPNB1,KRT1,PGM1,PPIA,PRDX6,PYGB,PYGL,RAP1B,SERPINE1,WDR1
Response to stimulus		51	7824	1.20 × 10^−5^	ACLY,ACTC1,ACTR3,AICDA,ALDOA,ANXA1,C1S,CDC42,CHIA,CLIC1,EEF1G,EXT1,F13A1,FAS,FBLN1,FBN1,FERMT3,FLNB,FSCN1,GNB2L1,GPC1,GPC6,GPRC5A,GSTP1,HMCN1,IGLL1,KPNB1,KRT1,LDHA,LOXL2,LRP1,LTBP1,MMP2,PDIA3,PGK1,PGM1,PHGDH,PLOD3,PPIA,PRDX2,PRDX6,PYGB,PYGL,RAP1B,SERPINE1,SULF1,SULF2,THBS4,UBA1,UBB,WNT5A
Negative regulation of cellular response to growth factor stimulus		8	137	1.59 × 10^−5^	FBN1,FBN2,GPC1,LTBP1,SULF1,SULF2,UBB,WNT5A
Immune system process		26	2370	2.26 × 10^−5^	ACLY,ACTR3,AICDA,ALDOA,ANXA1,C1S,CDC42,CHIA,FAS,FLNB,GPC1,GSTP1,IGLL1,KPNB1,KRT1,LRP1,PDIA3,PGM1,PPIA,PRDX6,PYGB,PYGL,RAP1B,UBB,WDR1,WNT5A
Carbohydrate metabolic process		12	457	2.26 × 10^−5^	ALDOA,AMY1B,CHIA,EXT1,FBN1,LDHA,LDHB,PGK1,PGM1,PYGB,PYGL,RGN
Immune response		21	1560	2.26 × 10^−5^	ACLY,ACTR3,AICDA,ALDOA,ANXA1,C1S,CHIA,FAS,FLNB,GSTP1,IGLL1,KPNB1,KRT1,LRP1,PGM1,PPIA,PRDX6,PYGB,PYGL,RAP1B,WNT5A
**KEGG Pathways**					
**Term Description**		**Obs**	**Bgr**	**FDR**	**Matching Proteins in the Network**
Glycolysis / Gluconeogenesis		5	68	0.00077	ALDOA,LDHA,LDHB,PGK1,PGM1
Proteoglycans in cancer		7	195	0.00077	CDC42,FAS,FLNB,FLNC,GPC1,MMP2,WNT5A
Focal adhesion		6	197	0.0035	CDC42,COMP,FLNB,FLNC,RAP1B,THBS4
Pentose phosphate pathway		3	30	0.0068	ALDOA,PGM1,RGN
Starch and sucrose metabolism		3	33	0.0071	PGM1,PYGB,PYGL
Metabolic pathways		13	1250	0.0095	ACLY,ALDOA,CHIA,EXT1,LDHA,LDHB,PGK1,PGM1,PHGDH,PRDX6,PYGB,PYGL,RGN
HIF-1 signaling pathway		4	98	0.0095	ALDOA,LDHA,PGK1,SERPINE1
Glucagon signaling pathway		4	100	0.0095	LDHA,LDHB,PYGB,PYGL
Malaria		3	47	0.0104	COMP,LRP1,THBS4
Carbon metabolism		4	116	0.011	ALDOA,PGK1,PHGDH,RGN
Fluid shear stress and atherosclerosis		4	133	0.0164	GPC1,GSTA5,GSTP1,MMP2
Biosynthesis of amino acids		3	72	0.0233	ALDOA,PGK1,PHGDH
Platinum drug resistance		3	70	0.0233	FAS,GSTA5,GSTP1
Necroptosis		4	155	0.0233	FAS,PPIA,PYGB,PYGL
Complement and coagulation cascades		3	78	0.0251	C1S,F13A1,SERPINE1
Salmonella infection		3	84	0.0288	CDC42,FLNB,FLNC
MAPK signaling pathway		5	293	0.0307	CDC42,FAS,FLNB,FLNC,RAP1B
AGE-RAGE signaling pathway in diabetic complications		3	98	0.0388	CDC42,MMP2,SERPINE1
Human papillomavirus infection		5	317	0.0388	CDC42,COMP,FAS,THBS4,WNT5A
Propanoate metabolism		2	32	0.0405	LDHA,LDHB
Leukocyte transendothelial migration		3	112	0.0476	CDC42,MMP2,RAP1B
Primary immunodeficiency		2	37	0.0481	AICDA,IGLL1
**10 Most Significant PMID Publications per FDR**					
**Term ID**	**Term Description**	**Obs**	**Bgr**	**FDR**	**Matching Proteins in the Network**
PMID:23823696	(2013) Isobaric Tagging-Based Quantification for Proteomic Analysis: A Comparative Study of Spared and Affected Muscles from mdx Mice at the Early Phase of Dystrophy.	8	42	1.26 × 10^−6^	ACLY,ALDOA,ANXA1,EEF1G,LDHB,PGM1,PPIA,PRDX2
PMID:29250190	(2017) Role of exosomes in hepatocellular carcinoma cell mobility alteration.	7	34	8.40 × 10^−6^	ANXA1,CLIC1,FBLN1,LRP1,PPIA,PYGB,PYGL
PMID:20140087	(2010) Comprehensive identification and modified-site mapping of S-nitrosylated targets in prostate epithelial cells.	9	103	9.47 × 10^−6^	ALDOA,ANXA1,CLIC1,FLNB,FLNC,KPNB1,PDIA3,PGK1,PLEC
PMID:29360750	(2018) Proteomic Analysis of Secretomes of Oncolytic Herpes Simplex Virus-Infected Squamous Cell Carcinoma Cells.	7	37	9.47 × 10^−6^	ACLY,ANXA1,FBN1,FLNC,FSCN1,MMP2,PRDX2
PMID:26779482	(2015) The Extracellular Matrix in Bronchopulmonary Dysplasia: Target and Source.	7	41	1.08 × 10^−5^	FBLN1,FBN1,FBN2,LOXL2,LTBP1,PLOD3,SULF2
PMID:24142637	(2013) Gastric autoantigenic proteins in Helicobacter pylori infection.	7	50	2.96 × 10^−5^	ACTR3,GSTP1,LDHB,PDIA3,PRDX2,PRDX6,WDR1
PMID:26184160	(2015) A Review: Proteomics in Nasopharyngeal Carcinoma.	8	83	2.96 × 10^−5^	ANXA1,CLIC1,KRT1,MMP2,PPIA,PRDX2,PRDX6,SERPINE1
PMID:26918450	(2016) A nuclear-directed human pancreatic ribonuclease (PE5) targets the metabolic phenotype of cancer cells.	8	89	3.71 × 10^−5^	ACLY,CLIC1,GPC1,GPC6,LDHA,PGM1,PHGDH,WNT5A
PMID:24223867	(2013) Lactate-modulated induction of THBS-1 activates transforming growth factor (TGF)-beta2 and migration of glioma cells in vitro.	6	31	5.98 × 10^−5^	COMP,LDHA,LDHB,MMP2,SERPINE1,THBS4
PMID:20236620	(2010) Unraveling the mechanism of elastic fiber assembly: The roles of short fibulins.	6	33	7.46 × 10^−5^	FBLN1,FBN1,FBN2,HMCN1,LOXL2,LTBP1

**Table 7:** PMIDs for the EV total unique proteins in the control group for the control vs. HIV comparison.

**The 10 Most Significant PMID Publications According to FDR**					
**Term ID**	**Term Description**	**Obs**	**Bgr**	**FDR**	**Matching Proteins in the Network**
PMID:19812696	(2009) Cancer genomics identifies regulatory gene networks associated with the transition from dysplasia to advanced lung adenocarcinomas induced by c-Raf-1.	3	154	0.0084	ACTC1,MAPT,MYH1
PMID:20587776	(2010) Mathematical modeling of endocytic actin patch kinetics in fission yeast: disassembly requires release of actin filament fragments.	2	12	0.0086	ACTC1,MYH1
PMID:25275480	(2014) Urethral dysfunction in female mice with estrogen receptor Beta deficiency.	2	10	0.0086	ACTC1,MYH1
PMID:22406440	(2012) Deferiprone reduces amyloid-Beta and tau phosphorylation levels but not reactive oxygen species generation in hippocampus of rabbits fed a cholesterol-enriched diet.	2	15	0.0088	ACTC1,MAPT
PMID:10931867	(2000) Distinct families of Z-line targeting modules in the COOH-terminal region of nebulin.	2	25	0.0099	ACTC1,MYH1
PMID:11994316	(2002) The NH2-terminal peptide of alpha-smooth muscle actin inhibits force generation by the myofibroblast in vitro and in vivo.	2	26	0.0099	ACTC1,MYH1
PMID:14557251	(2003) Skeletal myosin heavy chain function in cultured lung myofibroblasts.	2	26	0.0099	ACTC1,MYH1
PMID:17908293	(2007) Identification of genes differentially expressed during prenatal development of skeletal muscle in two pig breeds differing in muscularity.	2	52	0.0099	ACTC1,MYH1
PMID:19291799	(2009) Fast-twitch sarcomeric and glycolytic enzyme protein loss in inclusion body myositis.	2	36	0.0099	MAPT,MYH1
PMID:19325835	(2008) Myosin assembly, maintenance and degradation in muscle: Role of the chaperone UNC-45 in myosin thick filament dynamics.	2	44	0.0099	ACTC1,MYH1

**Table 8:** Biological processes, KEGG pathways, and PMIDs for the EV total unique proteins in the HIV group for the control vs. HIV comparison.

**Gene Ontology (GO) Terms for Biological Processes** **10 Most Significant Results per FDR (for All GO Terms, See Supplementary Table S4A)**					
**Term Description**		**Obs**	**Bgr**	**FDR**	**Matching Proteins in the Network**
Vesicle-mediated transport		57	1699	1.02 × 10^−18^	ACLY,ACTN1,ACTN4,ALDOA,ANXA1,ANXA11,ANXA5,AP2A1,AP2M1,APLP2,APOB,APOE,ARF3,ARF4,ARPC2,CAP1,CD44,CD59,CD63,CD81,EEF2,EHD1,EHD2,F13A1,FERMT3,FLNA,GAS6,ITIH3,ITIH4,KRT1,LAMP1,LAMP2,LOXL2,LRP1,MFGE8,MRC2,MVP,MYH9,PKP1,PRDX6,PTX3,RAB5C,RAB7A,RAC1,RAP1B,SERPINE1,SPTBN1,SRGN,SRPX,TGM2,THBS1,TLN1,TTN,UBB,VPS35,VWF,WDR1
Extracellular structure organization		28	339	7.06 × 10^−17^	AGRN,APOA4,APOB,APOE,BMP1,CD44,COMP,DCN,FBLN1,FBN1,GAS6,HTRA1,KLK7,LAMA1,LAMA2,LAMA4,LAMA5,LOXL2,MMP2,NID1,NID2,PLOD3,PXDN,SERPINE1,SULF1,SULF2,THBS1,VWF
Platelet degranulation		20	129	2.26 × 10^−16^	ACTN1,ACTN4,ALDOA,ANXA5,APLP2,CD63,F13A1,FERMT3,FLNA,GAS6,ITIH3,ITIH4,LAMP2,SERPINE1,SRGN,THBS1,TLN1,TTN,VWF,WDR1
Regulated exocytosis		35	691	1.19 × 10^−15^	ACLY,ACTN1,ACTN4,ALDOA,ANXA5,APLP2,CAP1,CD44,CD59,CD63,EEF2,F13A1,FERMT3,FLNA,GAS6,ITIH3,ITIH4,KRT1,LAMP1,LAMP2,MVP,PKP1,PRDX6,PTX3,RAB5C,RAB7A,RAC1,RAP1B,SERPINE1,SRGN,THBS1,TLN1,TTN,VWF,WDR1
Extracellular matrix organization		25	296	2.14 × 10^−15^	AGRN,BMP1,CD44,COMP,DCN,FBLN1,FBN1,GAS6,HTRA1,KLK7,LAMA1,LAMA2,LAMA4,LAMA5,LOXL2,MMP2,NID1,NID2,PLOD3,PXDN,SERPINE1,SULF1,SULF2,THBS1,VWF
Cellular component organization		89	5163	2.93 × 10^−14^	ACTN1,ACTN4,AGRN,ALDOA,ANXA1,ANXA6,AP2A1,AP2M1,APOA4,APOB,APOE,ARF4,ARPC2,ATL1,ATXN2,BMP1,CAP1,CD151,CD44,CD59,COMP,DCN,EHD1,EHD2,EXT1,FAS,FAT1,FBLN1,FBN1,FERMT3,FLNA,FLNB,FLNC,FSCN1,GAS6,GGCT,HIST1H4F,HTRA1,KLK7,KRT1,LAMA1,LAMA2,LAMA4,LAMA5,LAMP2,LOXL2,LTBP2,MFGE8,MMP2,MSRB1,MYH9,MYOF,NID1,NID2,PKP1,PLEC,PLOD3,PLS1,PLS3,PTGFRN,PXDN,RAB7A,RAC1,RAN,RHOC,SDC4,SEMG1,SERPINE1,SGCG,SLC25A6,SPAG1,SPTBN1,SRGN,SRPX,SULF1,SULF2,TGM1,TGM2,TGM3,THBS1,THY1,TLN1,TPM4,TTN,UBB,VPS35,VWF,WDR1,WNT5A
Secretion by cell		37	959	2.43 × 10^−13^	ACLY,ACTN1,ACTN4,ALDOA,ANXA1,ANXA5,APLP2,CAP1,CD44,CD59,CD63,EEF2,F13A1,FERMT3,FLNA,GAS6,ITIH3,ITIH4,KRT1,LAMP1,LAMP2,LTBP2,MVP,PKP1,PRDX6,PTX3,RAB5C,RAB7A,RAC1,RAP1B,SERPINE1,SRGN,THBS1,TLN1,TTN,VWF,WDR1
Response to stimulus		107	7824	6.96 × 10^−12^	ACLY,ACTN4,AFP,AGRN,AHCY,AICDA,ALDOA,ANXA1,ANXA11,ANXA5,ANXA6,AP2A1,AP2M1,APLP2,APOA4,APOB,APOE,ARF4,ARPC2,AZGP1,C1S,CAP1,CD151,CD44,CD59,CD63,CD81,CD82,CLIC1,DCN,EEF2,EHD1,EHD2,EXT1,EXT2,F13A1,FAS,FBLN1,FBN1,FERMT3,FLNA,FLNB,FSCN1,GAS6,GGCT,GNAI2,GNB2L1,GPC6,GPRC5A,HIST1H4F,HSPA5,ITIH4,KRT1,LAMA1,LAMA2,LAMA5,LAMP1,LAMP2,LDHA,LOXL2,LRP1,LTBP1,LTBP2,MMP2,MRC2,MSRB1,MVP,MYH9,MYOF,NNMT,PDIA3,PGK1,PKP1,PLOD1,PLOD3,POLR3G,PRDX1,PRDX6,PTX3,PXDN,RAB5C,RAB7A,RAC1,RAN,RAP1B,RHOC,SDC4,SEMG1,SERPINE1,SLC25A6,SPTBN1,SRGN,SRPX,STK33,SULF1,SULF2,TGM2,THBS1,THRB,THY1,TLN1,TTN,UBA1,UBB,VPS35,VWF,WNT5A
Localization		83	5233	9.26 × 10^−11^	ACLY,ACTN1,ACTN4,AGRN,ALDOA,ANXA1,ANXA11,ANXA5,ANXA6,AP2A1,AP2M1,APLP2,APOA4,APOB,APOE,ARF3,ARF4,ARPC2,ATXN2,AZGP1,CAP1,CD151,CD44,CD59,CD63,CD81,CLIC1,EEF2,EHD1,EHD2,F13A1,FAT1,FBN1,FERMT3,FLNA,FLNB,FSCN1,GAS6,GPC6,HSPA5,ITIH3,ITIH4,KRT1,LAMA5,LAMP1,LAMP2,LOXL2,LRP1,LTBP1,LTBP2,MFGE8,MRC2,MVP,MYH9,PKP1,PLOD3,PLS1,PRDX6,PTX3,RAB5C,RAB7A,RAC1,RAN,RAP1B,RHOC,SDC4,SERPINE1,SLC25A6,SPTBN1,SRGN,SRPX,SRPX2,TGM2,THBS1,THY1,TLN1,TTN,TTYH3,UBB,VPS35,VWF,WDR1,WNT5A
Anatomical structure development		80	5085	5.90 × 10^−10^	ACTN1,AEBP1,AFP,AGRN,AICDA,ANXA1,AP2A1,APOA4,APOB,APOE,ARF4,ATL1,BMP1,C6orf58,CAP1,CD151,CD44,COMP,DCN,EEF2,EHD1,EXT1,EXT2,FAS,FAT1,FBLN1,FBN1,FERMT3,FLNA,FLNB,FLNC,FSCN1,GAS6,GNB2L1,HSPA5,HTRA1,KLK7,KRT1,LAMA2,LAMA5,LDHA,LOXL2,LRP1,LTBP1,MFGE8,MMP2,MYH9,MYL6,MYOF,NID1,NNMT,PGK1,PKP1,PLOD1,PLOD3,PLS3,PPIB,PRDX1,RAC1,RAP1B,RHOC,SDC4,SERPINE1,SGCG,SPTBN1,SRGN,SRPX2,SULF1,SULF2,TGM1,TGM2,TGM3,THBS1,THBS3,THRB,THY1,TTN,UBB,WDR1,WNT5A
**KEGG Pathways**					
**Term Description**		**Obs**	**Bgr**	**FDR**	**Matching Proteins in the Network**
Focal adhesion		17	197	1.23 × 10^−10^	ACTN1,ACTN4,COMP,FLNA,FLNB,FLNC,LAMA1,LAMA2,LAMA4,LAMA5,RAC1,RAP1B,THBS1,THBS2,THBS3,TLN1,VWF
ECM-receptor interaction		12	81	5.47 × 10^−10^	AGRN,CD44,COMP,LAMA1,LAMA2,LAMA4,LAMA5,SDC4,THBS1,THBS2,THBS3,VWF
Proteoglycans in cancer		12	195	3.88 × 10^−6^	CD44,CD63,DCN,FAS,FLNA,FLNB,FLNC,MMP2,RAC1,SDC4,THBS1,WNT5A
Phagosome		10	145	1.40 × 10^−5^	COMP,LAMP1,LAMP2,MRC2,RAB5C,RAB7A,RAC1,THBS1,THBS2,THBS3
Amoebiasis		8	94	4.01 × 10^−5^	ACTN1,ACTN4,LAMA1,LAMA2,LAMA4,LAMA5,RAB5C,RAB7A
Malaria		6	47	8.88 × 10^−5^	CD81,COMP,LRP1,THBS1,THBS2,THBS3
Salmonella infection		7	84	0.00016	ARPC2,FLNA,FLNB,FLNC,MYH9,RAB7A,RAC1
Endocytosis		10	242	0.00054	AP2A1,AP2M1,ARF3,ARPC2,EHD1,EHD2,RAB5C,RAB7A,UBB,VPS35
Leukocyte transendothelial migration		7	112	0.0007	ACTN1,ACTN4,GNAI2,MMP2,RAC1,RAP1B,THY1
Human papillomavirus infection		11	317	0.00079	COMP,FAS,LAMA1,LAMA2,LAMA4,LAMA5,THBS1,THBS2,THBS3,VWF,WNT5A
PI3K-Akt signaling pathway		10	348	0.0069	COMP,LAMA1,LAMA2,LAMA4,LAMA5,RAC1,THBS1,THBS2,THBS3,VWF
Complement and coagulation cascades		5	78	0.0069	C1S,CD59,F13A1,SERPINE1,VWF
Cholesterol metabolism		4	48	0.0088	APOA4,APOB,APOE,LRP1
Toxoplasmosis		5	109	0.0226	GNAI2,LAMA1,LAMA2,LAMA4,LAMA5
Glycolysis / Gluconeogenesis		4	68	0.0259	ALDOA,LDHA,LDHB,PGK1
p53 signaling pathway		4	68	0.0259	CD82,FAS,SERPINE1,THBS1
Platelet activation		5	123	0.0308	FERMT3,GNAI2,RAP1B,TLN1,VWF
**10 Most Significant PMID Publications per FDR**					
**Term ID**	**Term Description**	**Obs**	**Bgr**	**FDR**	**Matching Proteins in the Network**
PMID:29250190	(2017) Role of exosomes in hepatocellular carcinoma cell mobility alteration.	17	34	1.84 × 10^−18^	ACTN1,ANXA1,ANXA11,ANXA5,ANXA6,APOB,APOE,CAP1,CLIC1,FBLN1,FLNA,ITIH4,LRP1,MFGE8,NID1,RAN,TLN1
PMID:24009881	(2012) Quantitative proteomics of extracellular vesicles derived from human primary and metastatic colorectal cancer cells.	21	161	9.74 × 10^−14^	AHCY,ANXA1,ANXA11,ANXA5,ANXA6,ARF3,ARPC2,CD44,CD63,CD81,FSCN1,KRT1,LAMP1,MFGE8,MYH9,MYL6,PGK1,PTGFRN,RAB5C,RAB7A,VPS35
PMID:19948009	(2009) Proteomic analysis of blastema formation in regenerating axolotl limbs.	22	221	1.76 × 10^−12^	ANXA1,ANXA11,ANXA5,ANXA6,DCN,EEF2,FBN1,FLNB,GNB2L1,MVP,MYH9,MYL6,MYOF,PDIA3,PLS3,PRDX1,PXDN,RAN,SND1,TTN,UBA1,WNT5A
PMID:24392111	(2014) Proteomic analysis of C2C12 myoblast and myotube exosome-like vesicles: a new paradigm for myoblast-myotube cross talk?	16	79	1.87 × 10^−12^	ALDOA,ANXA5,CD44,CD63,CD81,CD82,EEF2,FLNC,LAMP1,LAMP2,LDHA,MYOF,PGK1,TLN1,TTN,VPS35
PMID:27605433	(2016) Secreted primary human malignant mesothelioma exosome signature reflects oncogenic cargo.	17	107	5.36 × 10^−12^	ACLY,ANXA1,ANXA6,CD44,CD63,CD81,CD82,FAT1,GNB2L1,LAMA1,LAMP1,MFGE8,MMP2,PLS3,SULF1,THBS1,VPS35
PMID:22897585	(2012) Rat mammary extracellular matrix composition and response to ibuprofen treatment during postpartum involution by differential GeLC-MSMS analysis.	13	42	1.24 × 10^−11^	AGRN,ANXA1,ANXA11,ANXA5,ANXA6,CD44,DCN,FBN1,LAMA1,LAMA2,LAMA4,LAMA5,VWF
PMID:27770278	(2017) Comprehensive proteome profiling of glioblastoma-derived extracellular vesicles identifies markers for more aggressive disease.	14	63	3.75 × 10^−11^	ACTN4,ANXA1,CCT6A,CD44,EHD1,HSPA5,LAMA4,MMP2,MVP,MYH9,RAB5C,RAB7A,UBA1,VPS35
PMID:22159717	(2012) The matrisome: in silico definition and in vivo characterization by proteomics of normal and tumor extracellular matrices.	14	64	3.97 × 10^−11^	AGRN,ANXA1,ANXA11,ANXA5,ANXA6,DCN,FBN1,LOXL2,LTBP2,NID1,NID2,SRPX,THBS1,VWF
PMID:25201077	(2015) Proteomics of apheresis platelet supernatants during routine storage: Gender-related differences.	16	106	5.20 × 10^−-11^	ACTN1,APOB,APOE,ARPC2,C1S,FERMT3,FLNA,ITIH4,LDHA,MMP2,MYL6,PRDX6,SRGN,THBS1,TLN1,VWF
PMID:28071719	(2017) Quantitative proteomic profiling of the extracellular matrix of pancreatic islets during the angiogenic switch and insulinoma progression.	13	54	1.20 × 10^−10^	ANXA1,ANXA11,ANXA5,ANXA6,DCN,FBN1,LAMA1,LAMA2,LAMA4,LAMA5,NID1,NID2,THBS2

**Table 9:** Biological processes and PMIDs for the EV total unique proteins in the HIV group for the HIV vs. HIV+Aβ comparison.

**Gene ontology (GO) Terms for Biological Processes**					
**Term Description**		**Obs**	**Bgr**	**FDR**	**Matching Proteins in the Network**
Cell envelope organization		2	3	0.0017	TGM1,TGM3
**10 Most Significant PMID Publications per FDR**					
**Term ID**	**Term Description**	**Obs**	**Bgr**	**FDR**	**Matching Proteins in the Network**
PMID:22329734	(2012) Expression profile of cornified envelope structural proteins and keratinocyte differentiation-regulating proteins during skin barrier repair.	3	14	0.0016	KLK7,TGM1,TGM3
PMID:11093806	(2000) Transglutaminase-3, an esophageal cancer-related gene.	2	2	0.0136	TGM1,TGM3
PMID:11562168	(2001) Crystallization and preliminary X-ray analysis of human transglutaminase 3 from zymogen to active form.	2	2	0.0136	TGM1,TGM3
PMID:11980702	(2002) Three-dimensional structure of the human transglutaminase 3 enzyme: binding of calcium ions changes structure for activation.	2	2	0.0136	TGM1,TGM3
PMID:12850301	(2003) Analysis of epidermal-type transglutaminase (transglutaminase 3) in human stratified epithelia and cultured keratinocytes using monoclonal antibodies.	2	3	0.0136	TGM1,TGM3
PMID:14508061	(2003) A model for the reaction mechanism of the transglutaminase 3 enzyme.	2	2	0.0136	TGM1,TGM3
PMID:14645372	(2004) Structural basis for the coordinated regulation of transglutaminase 3 by guanine nucleotides and calciummagnesium.	2	2	0.0136	TGM1,TGM3
PMID:14987256	(2004) Identification of calcium-inducible genes in primary keratinocytes using suppression-subtractive hybridization.	2	8	0.0136	KLK7,TGM1
PMID:15084592	(2004) Crystal structure of transglutaminase 3 in complex with GMP: structural basis for nucleotide specificity.	2	2	0.0136	TGM1,TGM3
PMID:15172109	(2004) Transglutaminase activity and transglutaminase mRNA transcripts in gerbil brain ischemia.	2	3	0.0136	TGM1,TGM3

**Table 10:** Biological processes, KEGG pathways, and PMIDs for the EV total unique proteins in the HIV+Aβ group for the HIV vs. HIV+Aβ comparison.

**Gene Ontology (GO) Terms for Biological Processes** **10 Most Significant Results per FDR (for All GO Terms, See Supplementary Table S5A)**					
**Term Description**		**Obs**	**Bgr**	**FDR**	**Matching Proteins in the Network**
Vesicle-mediated transport		41	1699	2.01 × 10^−13^	ACTR3,AP1G1,AP2B1,ARF6,ARPC4,CALR,CAND1,CAPZB,CAV1,CD9,CDC42,COPB2,ECM1,EEA1,EHD4,IGF2R,KPNB1,MME,NME1,PDIA6,PGM1,PPIA,PSMD1,PSMD2,PSMD3,PYGB,PYGL,QSOX1,RAB10,RAB14,RAB1A,RAB2A,RALA,SLC44A2,SOD1,STOM,SYK,TGFB1,TIMP3,VAT1,XRCC6
Localization		66	5233	4.37 × 10^−11^	ACTR3,AP1G1,AP2B1,APOM,ARF6,ARPC4,CALR,CAND1,CAPZB,CAV1,CD9,CDC42,COPB2,CSE1L,DHX9,ECM1,EEA1,EHD4,FBN2,IGF2R,IGSF8,ILK,IPO5,IPO7,KPNB1,LMNA,MME,NME1,NRP1,PAFAH1B1,PDIA6,PGM1,PIP,PPIA,PSMD1,PSMD2,PSMD3,PYGB,PYGL,QSOX1,RAB10,RAB14,RAB1A,RAB2A,RALA,RELN,RNF128,RPL14,RTN4,SLC3A2,SLC44A1,SLC44A2,SLIT2,SOD1,SPOCK1,STOM,SYK,TGFB1,THBS4,TIMP3,VAT1,VDAC1,VDAC2,WLS,XPO1,XRCC6
Secretion		30	1070	8.12 × 10^−11^	CAND1,CAV1,CD9,ECM1,IGF2R,KPNB1,MME,NME1,PAFAH1B1,PGM1,PPIA,PSMD1,PSMD2,PSMD3,PYGB,PYGL,QSOX1,RAB10,RAB14,RAB1A,RALA,SLC44A2,SOD1,STOM,SYK,TGFB1,TIMP3,VAT1,WLS,XRCC6
Transport		57	4130	1.80 × 10^−10^	ACTR3,AP1G1,AP2B1,APOM,ARF6,ARPC4,CALR,CAND1,CAPZB,CAV1,CD9,CDC42,COPB2,CSE1L,DHX9,ECM1,EEA1,EHD4,IGF2R,IPO5,IPO7,KPNB1,LMNA,MME,NME1,NRP1,PAFAH1B1,PDIA6,PGM1,PIP,PPIA,PSMD1,PSMD2,PSMD3,PYGB,PYGL,QSOX1,RAB10,RAB14,RAB1A,RAB2A,RALA,RPL14,SLC3A2,SLC44A1,SLC44A2,SOD1,STOM,SYK,TGFB1,TIMP3,VAT1,VDAC1,VDAC2,WLS,XPO1,XRCC6
Secretion by cell		28	959	1.80 × 10^−10^	CAND1,CD9,ECM1,IGF2R,KPNB1,MME,PAFAH1B1,PGM1,PPIA,PSMD1,PSMD2,PSMD3,PYGB,PYGL,QSOX1,RAB10,RAB14,RAB1A,RALA,SLC44A2,SOD1,STOM,SYK,TGFB1,TIMP3,VAT1,WLS,XRCC6
Regulated exocytosis		24	691	2.67 × 10^−10^	CAND1,CD9,ECM1,IGF2R,KPNB1,MME,PGM1,PPIA,PSMD1,PSMD2,PSMD3,PYGB,PYGL,QSOX1,RAB10,RAB14,SLC44A2,SOD1,STOM,SYK,TGFB1,TIMP3,VAT1,XRCC6
Exocytosis		25	774	3.25 × 10^−10^	CAND1,CD9,ECM1,IGF2R,KPNB1,MME,PGM1,PPIA,PSMD1,PSMD2,PSMD3,PYGB,PYGL,QSOX1,RAB10,RAB14,RALA,SLC44A2,SOD1,STOM,SYK,TGFB1,TIMP3,VAT1,XRCC6
Neutrophil activation involved in immune response		19	489	1.06 × 10^−8^	CAND1,IGF2R,KPNB1,MME,PGM1,PPIA,PSMD1,PSMD2,PSMD3,PYGB,PYGL,QSOX1,RAB10,RAB14,SLC44A2,STOM,SYK,VAT1,XRCC6
Myeloid leukocyte activation		20	574	1.48 × 10^−8^	CAND1,IGF2R,KPNB1,MME,PGM1,PPIA,PSMD1,PSMD2,PSMD3,PYGB,PYGL,QSOX1,RAB10,RAB14,SLC44A2,STOM,SYK,TGFB1,VAT1,XRCC6
Neutrophil degranulation		18	485	4.95 × 10^−8^	CAND1,IGF2R,KPNB1,MME,PGM1,PPIA,PSMD1,PSMD2,PSMD3,PYGB,PYGL,QSOX1,RAB10,RAB14,SLC44A2,STOM,VAT1,XRCC6
**KEGG Pathways**					
**Term description**		**Obs**	**Bgr**	**FDR**	**Matching Proteins in the Network**
Endocytosis		10	242	0.00016	AP2B1,ARF6,ARPC4,CAPZB,CAV1,CDC42,EEA1,EHD4,IGF2R,RAB10
Focal adhesion		8	197	0.0012	CAV1,CDC42,ILK,LAMB2,PARVB,PPP1CB,RELN,THBS4
Bacterial invasion of epithelial cells		5	72	0.003	ARPC4,CAV1,CDC42,ILK,SEPT2
Pentose phosphate pathway		3	30	0.0278	G6PD,PGM1,RGN
Starch and sucrose metabolism		3	33	0.0278	PGM1,PYGB,PYGL
Proteoglycans in cancer		6	195	0.0278	CAV1,CDC42,LUM,PPP1CB,TGFB1,TIMP3
Proteasome		3	43	0.0347	PSMD1,PSMD2,PSMD3
Necroptosis		5	155	0.0347	PPIA,PYGB,PYGL,VDAC1,VDAC2
Fc gamma R-mediated phagocytosis		4	89	0.0347	ARF6,ARPC4,CDC42,SYK
Amino sugar and nucleotide sugar metabolism		3	48	0.0396	CHIA,PGM1,UGDH
HTLV-I infection		6	250	0.0396	CALR,NRP1,TGFB1,VDAC1,VDAC2,XPO1
**10 Most Significant PMID Publications per FDR**					
**Term ID**	**Term Description**	**Obs**	**Bgr**	**FDR**	**Matching Proteins in the Network**
PMID:11149929	(2001) The phagosome proteome: insight into phagosome functions.	9	47	3.12 × 10^−6^	ARF6,CALR,DFFA,P4HB,RAB10,RAB14,RAB2A,STOM,VDAC1
PMID:17892558	(2007) Quantifying raft proteins in neonatal mouse brain by ‘tube-gel’ protein digestion label-free shotgun proteomics.	10	83	6.99 × 10^−6^	ACTC1,BASP1,CAV1,CNTN1,RAB10,RAB14,RAB1A,RAB2A,SLC3A2,VDAC1
PMID:22578496	(2012) Harnessing the power of the endosome to regulate neural development.	7	35	0.00014	ARF6,EEA1,EHD4,NRP1,RAB14,RTN4,WLS
PMID:24009881	(2012) Quantitative proteomics of extracellular vesicles derived from human primary and metastatic colorectal cancer cells.	11	161	0.00014	ACTR3,CAPZB,CD9,EHD4,ILK,RAB10,RALA,SLC3A2,SLC44A1,SYK,UGDH
PMID:27770278	(2017) Comprehensive proteome profiling of glioblastoma-derived extracellular vesicles identifies markers for more aggressive disease.	8	63	0.00016	ACTR3,CALR,ECM1,IGF2R,IPO5,PSMD2,RAB10TGFB1
PMID:26205348	(2015) Fluoxetine increases plasticity and modulates the proteomic profile in the adult mouse visual cortex.	6	22	0.00023	AP1G1,CDC42,NME1,SOD1,VDAC1,VDAC2
PMID:20140087	(2010) Comprehensive identification and modified-site mapping of S-nitrosylated targets in prostate epithelial cells.	9	103	0.00024	DHX9,HNRNPK,KPNB1,LMNA,P4HB,PDIA6,RTN4,VDAC1,VDAC2
PMID:27549615	(2016) Genome-wide association study to identify potential genetic modifiers in a canine model for Duchenne muscular dystrophy.	6	23	0.00024	LMNA,PAMR1,PPIA,PSMD2,SLIT2,THBS4
PMID:23170974	(2012) Integrated miRNA, mRNA and protein expression analysis reveals the role of post-transcriptional regulation in controlling CHO cell growth rate.	6	27	0.00044	HNRNPK,RAB10,RAB14,RAB1A,RAB2A,RPL14
PMID:24505448	(2014) Characterisation of four LIM protein-encoding genes involved in infection-related development and pathogenicity by the rice blast fungus Magnaporthe oryzae.	6	28	0.00047	CDC42,ILK,LMNA,PHGDH,RAB2A,XRCC6

## Supplementary Materials

The following are available online at https://www.mdpi.com/1422-0067/21/8/2741/s1.

## Abbreviations

Aβ	amyloid beta
AD	Alzheimer’s disease
BBB	Blood–brain barrier
ECGS	Endothelial cell growth supplement
EV	Extracellular vesicle
ELISA	Enzyme-linked immunosorbent assay
HAND	HIV-associated neurocognitive disorders;
HBMEC	Human brain microvascular endothelial cells
HEK cells	Human embryonic kidney cells
HIV	Human immunodeficiency virus type 1
PBS	Phosphate buffered saline
PECAM	Platelet endothelial cell adhesion molecule
RAGE	Receptor for advanced glycation end products
